# Lateral inhibition governs ancestral cellular patterning in fossil and extant liverworts

**DOI:** 10.1242/dev.205696

**Published:** 2026-07-23

**Authors:** Josep Mercadal, Susan Tremblay, Leonie Kraska, Martin A. Hutten, Pau Formosa-Jordan

**Affiliations:** ^1^ Department of Plant Developmental Biology, Max Planck Institute for Plant Breeding Research, 50829 Cologne, Germany; ^2^ Department of Research and Collections, New York State Museum, Madison Avenue, Albany, NY 12230, USA; ^3^ LadHyX, CNRS, École Polytechnique, Institut Polytechnique de Paris, Palaiseau Cedex 91128, France; ^4^ DOI National Park Service, Glacier Bay National Park & Preserve, Gustavus, AK 99826, USA; ^5^ Cluster of Excellence on Plant Sciences (CEPLAS), Max Planck Institute for Plant Breeding Research, 50829 Cologne, Germany

**Keywords:** Lateral inhibition, Fossils, Liverworts, Oil body cells, Pattern formation, Evo-devo

## Abstract

The incompleteness of the fossil record and the absence of genetic information limit our ability to understand ancient developmental processes and reconstruct their evolution. Gene expression patterns, physiological pathways, or cellular differentiation processes are rarely preserved, leaving major gaps in our understanding of developmental evolution. Here, we combine comparative and mathematical analyses to uncover an ancestral patterning mechanism underlying the specification of liverwort oil body cells, a synapomorphy of the lineage involved in anti-herbivory defense. We demonstrate that oil body cell homologs in fossils of the oldest known liverwort (*Metzgeriothallus sharonae*) exhibit spatial organization characteristic of lateral inhibition, including regular spacing and an over-representation of idioblasts. We show that a mechanism involving local activation and lateral inhibition reproduces the diversity of patterns observed in the fossils. Finally, we find that the same mechanism can explain oil body cell patterns in the distantly related liverworts *Treubia lacunosa*, *Apotreubia nana*, and *Marchantia polymorpha*, suggesting a deeply conserved mode of cell specification. Our findings illuminate the origin and diversification of oil body cells, underscoring their evolutionary significance for liverwort development and survival.

## Teaser

**Highlighted Article:** Comparative and mathematical analyses reveal that oil body cell patterning in fossil and extant liverworts is consistent with a local activation-lateral inhibition mechanism, pointing to deep conservation of this patterning mechanism since the Devonian.

## INTRODUCTION

Evolutionary developmental biology has historically relied on comparative anatomy, morphometrics and systematics, with fossil evidence playing a crucial role in inferring developmental patterns and their evolutionary relationships (Hall, 2002; Gilbert, 2003; Amundson, 2005). While modern approaches to understanding the evolution of development have gravitated towards molecular techniques, these remain inadequate when confronted with ancient fossil data, as genetic material older than approximately 1 Ma is rarely preserved, with the oldest DNA recovered from mammoth specimens dating to around 1 Ma (Van Der Valk et al., 2021) and the oldest environmental DNA dating to around 2 Ma (Kjær et al., 2022), thereby leaving the vast majority of the fossil record inaccessible. Consequently, reconstructing dynamical aspects of development, such as morphogenesis and cell-specification mechanisms, remains challenging and often underexplored. Recent advances in imaging and mathematical modeling have revived comparative approaches (Alberch, 1991; Sepkoski, 2005; Cunningham et al., 2014; Jaeger and Monk, 2014; DiFrisco and Jaeger, 2021), making it possible to recognize morphological fingerprints of developmental mechanisms in living and extinct organisms (Sanders et al., 2007; Chipman and Edgecombe, 2019; Rothwell and Tomescu, 2021; Haug and Haug, 2021; Tomescu and Rothwell, 2022). In animals, these include the evolution of tooth development (Salazar-Ciudad and Jernvall, 2010; Harjunmaa et al., 2014; Ortiz et al., 2018; Zimm et al., 2023), the fin-to-limb transition (Raspopovic et al., 2014; Onimaru et al., 2016; Stewart et al., 2020), and patterning of epidermal structures (Cooper et al., 2018; Staps et al., 2023; Tseng et al., 2024), among others. Although less studied in this context, plants offer a unique opportunity to investigate ancestral developmental mechanisms, as preserved cell walls and fixed cell positions facilitate the recovery of developmentally informative anatomy (Boyce, 2008; Delaux et al., 2019; Petrone-Mendoza et al., 2023; Hetherington et al., 2016; Hetherington, 2024). Evidence includes the presence of ancient polar auxin transport from growth patterns in wood and vascular tissues (Rothwell and Lev-Yadun, 2005; Rothwell et al., 2008), or polyploidy and conserved patterning in fossil stomata (Masterson, 1994; McElwain and Steinthorsdottir, 2017; Rudall and Bateman, 2019).

A striking example of well-preserved cellular patterning can be observed in the Middle Devonian (∼388 Ma) fossil *Metzgeriothallus sharonae* (Hernick et al., 2008), the oldest known liverwort. A defining feature of these fossils is the presence of dark, scattered cells that closely resemble oil body (OB) cells of modern liverworts (Fig. 1A), suggesting evolutionary homology (Schuster, 1966; Tremblay and Mercadal, 2026) and implicating these structures in the chemical defense of liverworts against arthropod herbivory (Labandeira et al., 2014). Dark cells are distributed more or less evenly throughout the thallus wings, often forming characteristic ‘salt-and-pepper’ patterns (Fig. 1). This spatial arrangement is associated with the phenomenon of lateral inhibition, whereby adjacent cells compete to differentiate into distinct types by inhibiting the same fate as their neighbors, generating regularly spaced patterns (Collier et al., 1996; Cohen et al., 2010; Guisoni et al., 2017).

Through spatial statistics, we show that dark cell patterns in *M. sharonae* exhibit a distinctive spatial regularity characterized by an over-representation of isolated dark cells and negative spatial autocorrelation. We demonstrate that these patterns are consistent with cell specification mediated by local activation and lateral inhibition, accounting for the full diversity of patterns observed in fossil samples. Finally, we show that the same cell specification mechanism can reproduce the spatial organization of OB cells in the extant liverworts *Treubia lacunosa*, *Apotreubia nana*, and *Marchantia polymorpha*, supporting the evolutionary conservation of an ancestral patterning process. Our framework explains lineage-specific phenotypic transitions, such as the complete loss of OBs or the shift from ubiquitous to idioblastic OB distribution, as outcomes of a conserved patterning module being repurposed over evolutionary time.

## RESULTS

### Dark cell pattern diversity and spatial statistics

We collected *M. sharonae* material from the Cairo quarry in the Catskill Delta deposit of eastern New York state, USA, well-known for its diverse macroflora, and the only known liverwort-dominated community from the Paleozoic (Hernick et al., 2008; Labandeira et al., 2014; Tremblay and Mercadal, 2026). From a total of 120 collected *M. sharonae* samples, we selected 58 sections based on the size (>1 mm^2^), good preservation of cellular structures, and clearly defined dark cells. The total number of cells in each section ranged from approximately 400 to 900 cells. Individual specimens typically had a homogeneous density of dark cells in the wings of thalli (Fig. 1A-C), although most were fragmented and preserved only 2-3 cm of a single thallus. Some regions interpreted as growing tissues, such as apices, bases of developing branches, and small branches emerging from the midribs of mature thalli, have recently been shown to exhibit a higher frequency of dark cells (Tremblay and Mercadal, 2026), possibly reflecting a protective role against herbivory in these developing tissues (Labandeira et al., 2014). Occasionally, certain regions within a thallus wing exhibited a higher frequency of dark cells (Fig. 1D), which have been interpreted as areas affected by microbial attacks, desiccation, or other forms of environmental stress (Labandeira et al., 2014; Tremblay and Mercadal, 2026). In contrast, some specimens exhibited only sparse distributions of dark cells or lacked them entirely (Fig. 1E). Owing to the variability in frequency and regularity of dark cell patterns, we classified sections into four categories: (I) regular patterns with high dark cell frequency (*ρ*_*I*_ ≥ 0.19), where dark cells formed salt-and-pepper patterns with a separation of one or two epidermal cells (Fig. 1B); (II) regular patterns with low dark cell frequency (*ρ*_*II*_ < 0.19), where dark cells were separated by two to five epidermal cells (Fig. 1C); (III) mixed patterns, where the regularity of dark cell patterns was not obvious due to the presence of gaps and large clusters (Fig. 1D); and (IV) patterns with very few scattered dark cells (Fig. 1E). Most sections belonged to category II (*n*_*II*_=43), whereas only a few belonged to categories I (*n*_*I*_=7), III (*n*_*III*_=5) and IV (*n*_*IV*_=3). Because dark cells were rare in type IV sections, we excluded them from the statistical analyses, leaving a total of *n*=55 fossil sections.

**Fig. 1.**
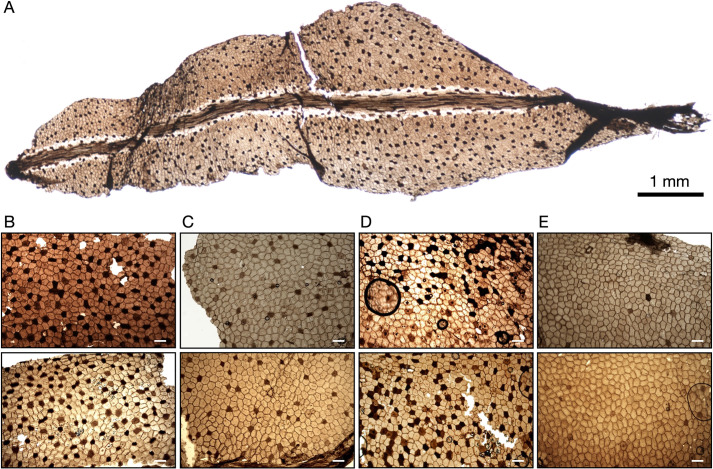
**Light micrographs of the Middle Devonian fossil liverwort *Metzgeriothallus sharonae*.** (A) Thallus comprising a midrib and unistratose wings with scattered dark cells. Scale bar: 1 mm. (B-E) Representative images of *M. sharonae* showing sections of different thalli, in which scattered dark cells can be readily discerned. These dark cells are regularly distributed across space, mostly isolated (idioblasts) or forming small clusters of two to three cells (B,C). Occasionally, larger, mostly linear clusters of dark cells can also be observed (D). In a few sections, dark cells appear only occasionally and in idioblastic form (E). Scale bars: 0.1 mm. Specimen accession numbers are: UC250151.14 (B, top), UC250150.12 (B, bottom), UC250155.27 (C, top), UC250153.07 (C, bottom), UC250156.21 (D, top), UC250152.05 (D, bottom), UC250151.17 (E, top), and UC250155.09 (E, bottom). The specimen in A could not be preserved to become a permanent slide.

We used the following metrics to characterize the spatial distribution of dark cells (Materials and Methods; Figs S1 and S2): dark cell frequency *ρ*, i.e. the proportion of dark cells relative to the total number of cells in the tissue; the distribution of sizes of dark cell clusters *f*_*k*_, where *k* represents the cluster size; the distribution of cell distances between pairs of neighboring dark cells *D*_*j*_, where *j* stands for the number of separating epidermal cells; and the spatial autocorrelation via Moran's *I* statistic. We compared these metrics with those obtained with a random model of cell specification, in which each cell becomes dark with probability *p* or epidermal with probability 1−*p* (Fig. 2; Fig. S3). We performed simulations of the random model on irregular cell lattices with cell numbers similar to those in fossil samples (Fig. 2A). In fossils, dark cells appeared with a frequency *ρ* = 0.16±0.2 (Fig. 2A,B). The distribution of cluster sizes showed a strong excess of idioblasts (clusters of size *k* = 1, i.e. isolated dark cells surrounded by light cells; Fig. S2D), significantly more than what would be expected by random specification (Fig. 2C,D). In contrast, larger clusters occurred at considerably lower proportions than expected by chance (Fig. 2D) and typically took the form of linear arrays, which are distinctive hallmarks of lateral inhibition (Thamm et al., 2020; Mercadal et al., 2026). Most dark cells were separated by one to three cells, with an average of ≈2.7 cells (Fig. 2E). This regularity was in contrast to the random model, in which dark cell separation was significantly more variable (Fig. 2E; Fig. S2).

**Fig. 2.**
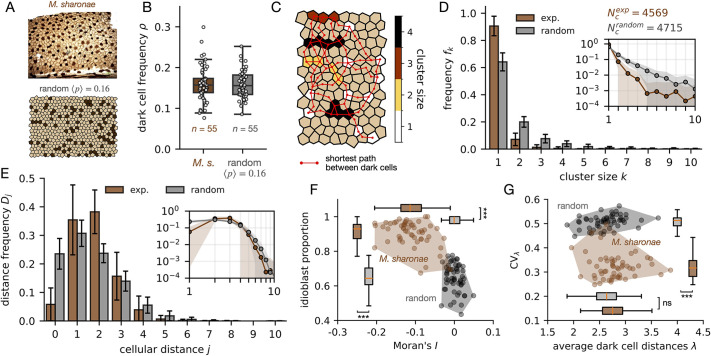
**Non-randomness of dark cell patterns in *M. sharonae* fossils.** (A) Representative section of *M. sharonae* showing a dark cell pattern (top; UC250150.12, same as Fig. 1B bottom) and a simulated random pattern with similar dark cell frequency (bottom). (B) Boxplots showing the frequency of dark cells for fossil sections (brown; *n*=55) and for random patterns (gray; *n*=55) with dark cell probability *p* = 0.16 × (1 + 0.7
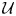
[−0.5, 0.5]), where 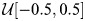 denotes a uniform distribution of random numbers between −0.5 and 0.5. With this choice, random patterns exhibit a frequency distribution similar to that of fossil samples. (C) Metrics used to determine the spatial statistics of dark cell patterns, including the cluster sizes in different colors and the shortest paths between neighboring dark cells in red (see Fig. S1 and Materials and Methods for a complete description of these metrics). (D) Distribution of cluster sizes in fossil patterns (brown) compared to random patterns (gray). The inset shows the same data on a log-log scale. *N*_*c*_ stands for the total number of clusters analyzed. (E) Distribution of distances (shortest paths) between dark cells in fossils (brown) and random patterns (gray). The inset shows the same data on a log-log scale. (F) Morphospace showing the proportion of idioblastic dark cells versus Moran's *I* in fossil (brown) and random (gray) patterns. (G) Morphospace showing the average distances between dark cells versus their corresponding coefficient of variation of fossil (brown) and random (gray) patterns. In B, F, and G, box limits represent interquartile ranges (IQR=Q3−Q1), orange lines represent medians, and whiskers extend from Q1−1.5×IQR to Q3+1.5×IQR. Error bars and shaded areas in D and E denote standard deviations (s.d.). The shaded regions in F and G represent the convex hulls of each dataset. Statistical significance was assessed using the Mann–Whitney *U*-test; ***P<0.01; ns, non-significant.

To better understand how dark cell patterns compare to a random model of cell specification, we located each fossil sample in the morphospaces of Moran's *I* statistic versus idioblast proportion, and average dark cell distance 〈*λ*〉 versus 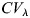, the coefficient of variation of these distances (see Materials and Methods for how these metrics are defined). Our results showed that, in these spaces, dark cell patterns cluster without overlapping with random patterns (Fig. 2F,G). Dark cell patterns had a high proportion of idioblasts and negative values of Moran's *I*, revealing a spatial organization that the random model cannot reproduce (Fig. 2F). While the average distance between dark cells was similar between the fossils and the random model, the 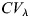 was significantly lower in the fossils (Fig. 2G), reflecting lower variability in these distances and a characteristic pattern regularity. Altogether, these results show that the statistical properties of dark cell patterns in fossils are incompatible with random cell specification. This suggests that the mechanism of dark cell specification in *M. sharonae* was not random, as would be expected by a cell-autonomous process, but likely involved cell-to-cell interactions via lateral inhibition, reducing the occurrence of adjacent dark cells and generating the observed spatial regularity.

### A model with local activation and lateral inhibition reproduces the diversity of dark cell patterns

Developmental mechanisms generating cell type-specific patterning typically rely on cell-to-cell communication through variants of lateral inhibition (Salazar-Ciudad et al., 2003; Torii, 2012; Schweisguth and Corson, 2019). While the genetic and molecular factors involved in patterning might differ significantly across biological systems, the dynamics and spatial statistics are often telltale signs of the underlying developmental processes and offer crucial information for distinguishing between mechanisms (Hiscock and Megason, 2015; Summers et al., 2022). Owing to the lack of genetic and dynamical information, we compared the spatial statistics of dark cell patterns in *M. sharonae* with those obtained with a minimal cellular model of pattern formation, where a local activator *u* induces the activity of a mobile inhibitor *v* (Meinhardt and Gierer, 2000; Mercadal et al., 2026) (Fig. 3A; Materials and Methods). At each time point, the state of each cell is described by the values of *u*(*t*) and *v*(*t*). We assume the activator is constantly produced, self-activates, and is degraded linearly and by the inhibitor (Fig. 3B). The inhibitor is also constantly produced, is induced by the activator, is linearly degraded, and can diffuse to adjacent cells (Fig. 3B). We explored the steady-state patterning properties of the model in an irregular cellular tissue by varying the parameters *γ* (the maximum rate at which *u* activates *v*) and *α*_0_ (the basal production rate of *u*) (Fig. 3C; Fig. S4). We used the stationary values of *u* as a proxy for cell fate, with high *u* indicating dark cells and low *u* indicating epidermal cells.

**Fig. 3.**
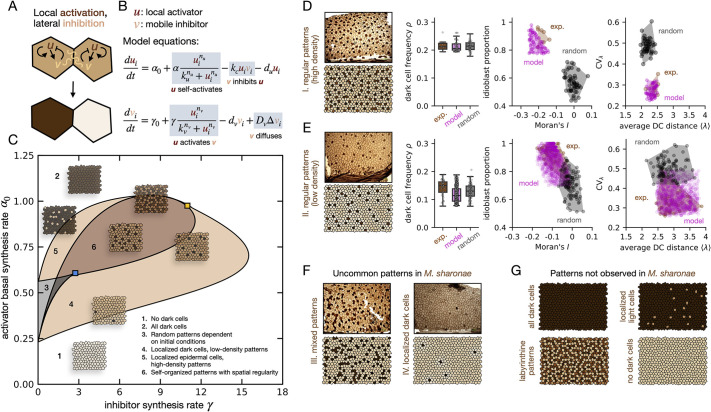
**A model with local activation and lateral inhibition reproduces the diversity of dark cell patterns in *M. sharonae* fossils.** (A) Schematic of the interactions in a cellular model of local activation and lateral inhibition. The state of two adjacent undifferentiated cells can be destabilized due to diffusive lateral inhibition, priming them to adopt opposite fates. (B) Equations of the mathematical model corresponding to the interactions depicted in A. The key regulations are highlighted in blue. (C) Stability diagram showing different patterning regimes obtained with the mathematical model as a function of the parameters *γ* and *α*_0_ (Materials and Methods; Fig. S4). The blue square denotes a cusp point at which two saddle-node bifurcations collide and annihilate; the yellow square denotes the collision between a subcritical Turing bifurcation and two saddle-node bifurcations. Snapshots of representative simulated patterns are displayed across the stability diagram. (D) Comparison of the spatial statistics of high-density fossil samples (exp., experimental; brown; *n*=7) with the mechanistic (pink; *n*=55) and random (gray; *n*=100) models (see Fig. S6). (E) Comparison of low-density fossil samples (brown; *n*=43) with the mechanistic (pink; *n*=826) and random (gray; *n*=100) models (see Fig. S6). (F) In a few sections, dark cells are inhomogeneously distributed (left) or appear only occasionally (right). Statistics were not performed due to the small number of sections of these types. Nevertheless, the mathematical model can qualitatively reproduce the corresponding patterns (Fig. S6). (G) Several patterns predicted by the model are not observed in the fossils. Cell colors represent the values of the variable *u* in each cell, in the interval *u*∈[0.2, 1.2] (see Fig. S4). The values of *α*_0_ and *γ* used for D-G are specified in Fig. S6. The rest of the parameters are detailed in Table S1. In D and E, box limits represent interquartile ranges (IQR=Q3−Q1), horizontal lines represent medians, and whiskers extend from Q1–1.5×IQR to Q3+1.5×IQR. Shaded areas in D and E denote the convex hulls of each dataset. Fossil images in D, E, and F are the same as the bottom images of Fig. 1B,C,D, and the top image of Fig. 1E (UC250150.12, UC250153.07, UC250152.05, and UC250151.17).

Analyzing the model through bifurcation theory and linear stability analysis (Collier et al., 1996; Strogatz, 2018) revealed different patterning regimes (Fig. 3C; Fig. S4). If *α*_0_ was small, the activator was not strong enough to induce cell differentiation, and no dark cells emerged (Fig. 3C, region 1). Conversely, for large *α*_0_, all cells became dark (region 2). Patterns with different dark cell frequencies and regularities emerged for intermediate values of (*γ*, *α*_0_). The system had a bistable region whereby cellular fates were strongly influenced by initial conditions (region 3). Here, lateral inhibition does not play a significant role because all possible cell combinations are stable (Fig. S4). The model can also generate localized patterns with different densities (regions 4 and 5) that are triggered only by specific initial conditions (Fig. S4). Finally, for a wide range of (*γ*, *α*_0_), the system could generate self-organized patterns (region 6), whereby ordered spatial patterns emerged spontaneously due to the amplification of inhomogeneous perturbations. Patterns across this region differed in their spatial statistics, including dark cell frequency, idioblast proportion, and average dark cell distance (Fig. S4). Mixed patterns, where periodic domains co-exist with more random-looking patterns, could be obtained when the values of (*γ*, *α*_0_) were close to the bistable region (regions 4, 5, and 6 close to region 3; Fig. S4). We obtained equivalent results using an alternative model in which inhibition occurs transcriptionally (Fig. S5), demonstrating that our findings are robust to the specific mechanistic implementation of inhibition, provided that the underlying regulatory logic is preserved.

We performed a parameter sweep over the pairs (*γ*, *α*_0_) to test whether the model could reproduce the statistical metrics of fossil patterns (dark cell frequency, idioblast proportion, Moran's *I*, average dark cell distance 〈*λ*〉, and 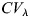) across the four categories of our classification (Fig. 3D,F; Fig. S6). For each category, we identified the pairs (*γ*, *α*_0_) that generated patterns in which all metrics simultaneously fell within their observed experimental ranges (Materials and Methods; Fig. S6). We found that the model could reproduce all the observed phenotypes, generating patterns in morphospace regions that virtually overlap or contain the experimental results (Fig. 3D,E). Notably, the model yielded multiple parameter pairs that satisfy all these conditions (Fig. S6), underscoring the mechanisms' ability to generate similar phenotypes through different routes. Uncommon patterns, such as those with no well-defined regularity or very few localized dark cells, could also be reproduced by the model (Fig. 3F; Fig. S6). Additionally, the model could generate phenotypes that are not observed in fossil samples. These included tissues completely covered in dark cells, tissues with mostly dark cells and few localized light cells, labyrinthine patterns, and tissues without dark cells (Fig. 3G; Figs S4, S6).

Taken together, these results demonstrate that a cell specification mechanism through local activation and lateral inhibition can explain the full variation of dark cell patterns in *M. sharonae*. This flexibility in generating diverse patterning phenotypes highlights the evolutionary potential of the mechanism, enabling adaptive responses to varying selective pressures.

### An evolutionarily conserved patterning mechanism for OB cell specification

Our results suggest a cell patterning mechanism underlying the specification of dark cells in *M. sharonae*, the oldest known fossil liverwort. Dark cells in *M. sharonae* and other Paleozoic liverworts are considered homologous to the OB cells of modern liverworts (Labandeira et al., 2014; Tremblay and Mercadal, 2026). Hence, to investigate whether this mechanism has been conserved across the liverwort lineage, we analyzed the spatial distribution of OB cells in thalli of the extant liverworts *T. lacunosa*, *A. nana*, and *M. polymorpha* (Fig. 4). In *T. lacunosa* and *A. nana*, cells harboring OBs were, on average, larger than non-OB cells, and most appeared as idioblasts (Fig. 4A,C). Notably, they occurred at a similar frequency as in *M. sharonae* (*ρ* ≈ 0.15; Fig. 4B,D). Most of the OB cell patterns in *T. lacunosa* and *A. nana* were inconsistent with random cell specification, despite some overlap in the regions occupied by the morphospaces (Fig. 4B,D). Instead, both the idioblast proportion, Moran's *I*, average OB cell distance 〈*λ*〉, and its coefficient of variation 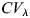 were consistent with patterns shaped by lateral inhibition. To test whether our model could reproduce the observed statistics, we looked for pairs (*γ*, *α*_0_) for which the statistical metrics reproduced OB cell patterns in *T. lacunosa* and *A. nana*. We found several pairs that fulfilled all the conditions (Fig. 4B,D, pink; Fig. S7), spanning a morphospace that virtually overlapped or contained the experimental results. Therefore, OB cell patterns in *T. lacunosa* and *A. nana* are consistent with cell specification governed by local activation and lateral inhibition.

**Fig. 4.**
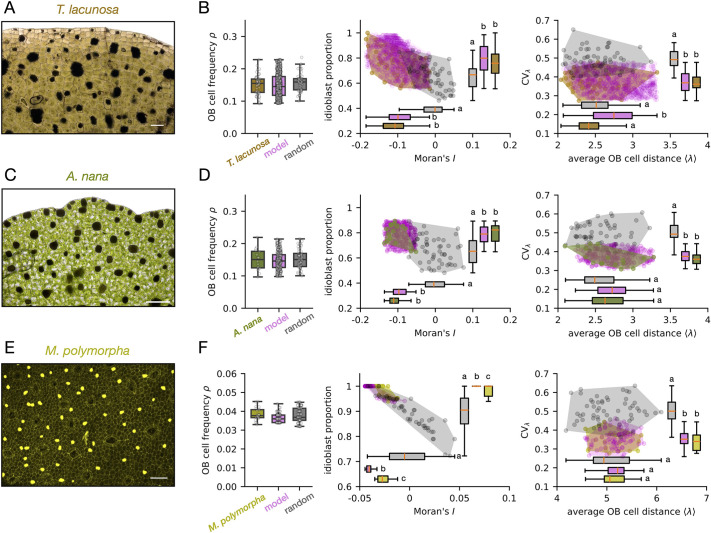
**Lateral inhibition patterning of OB cells is conserved across liverwort taxa.** (A) Distribution of OB cells in *T. lacunosa* (UC2036946). (B) Spatial statistics comparing experimental OB cell patterns in *T. lacunosa* (brown; *n*=50) with patterns obtained with the mechanistic (pink; *n*=777) and random (gray; *n*=55) models. (C) Distribution of OB cells in *A. nana*. (D) Spatial statistics comparing OB cell patterns in *A. nana* (green; *n*=16) with patterns obtained with the mechanistic (pink; *n*=305) and random (gray; *n*=55) models. (E) *M. polymorpha* thallus stained with BODIPY, exposing oil bodies and cell boundaries (Fig. S8). (F) Spatial statistics comparing OB cell patterns in *M. polymorpha* (gold; *n*=21) with patterns obtained with the mechanistic (pink; *n*=84) and random (gray; *n*=55) models. Box limits represent interquartile ranges (IQR=Q3–Q1), horizontal lines represent medians, and whiskers extend from Q1−1.5×IQR to Q3+1.5×IQR. Shaded areas denote the convex hulls of each dataset. Statistical differences in B, D, and F were tested using ANOVA followed by Tukey HSD (*P*<0.01); letters indicate statistically significant groups. Scale bars: 0.1 mm.

Finally, we tested whether our model could reproduce OB cell patterns in *M. polymorpha*. In *M. polymorpha* thalli, OB cells are typically located in the regions between air pores, suggesting that there is likely an interplay between air pore development and OB cell positioning. Nevertheless, OB cells appeared almost exclusively in idioblastic form, supporting the hypothesis that their specification is governed by lateral inhibition (Fig. 4E,F; Fig. S9). OB cell frequency in *M. polymorpha* was lower than in fossils, *T. lacunosa*, and *A. nana*, likely in part due to spatial exclusion by air pores (Fig. 4; Fig. S9). Consequently, the average distance between OB cells was higher than in the other species. By contrast, the coefficient of variation spanned the same scale, suggesting that, despite differences in frequency and OB cell distance, the pattern variability is conserved (Fig. 4B,D,F). Comparing these distributions with a random model that has the same OB cell frequency revealed that patterns in *M. polymorpha* are also consistent with a mechanism involving local activation and lateral inhibition. Indeed, our model reproduced all the spatial statistics associated with these patterns (Fig. 4F; Fig. S7).

Taken together, our results show that a mechanism involving local activation and lateral inhibition can reproduce the spatial patterns of OB cells not only in *M. sharonae* fossils, but also in the extant liverworts *T. lacunosa*, *A. nana*, and *M. polymorpha*. This consistency across distantly related liverwort taxa points to an as-yet-unidentified, deeply conserved developmental mechanism underlying liverwort OB cell specification.

## DISCUSSION

Oil bodies play important roles in lipid and secondary metabolite storage, thereby contributing significantly to plant defense mechanisms (He et al., 2013; Tanaka et al., 2016; Romani et al., 2022; Kanazawa et al., 2020; Schweizer et al., 2025). The regular and idioblastic nature of OB cells might reflect a strategy to minimize unnecessary overlap, potentially optimizing their protective or signaling functions. Despite their putative defensive role, the frequency and spatial distribution of OB vary among liverwort taxa. In Haplomitriopsida, taxa in the genus *Haplomitrium* have several OBs in all cells. In contrast, those in the Treubiales (like *T. lacunosa* and *A. nana*) have a single OB in idioblastic cells (Flores et al., 2018; Romani et al., 2022) (Fig. 5A). In Marchantiopsida, most taxa have a single OB in idioblastic cells, whereas in Jungermanniopsida (where *M. sharonae* is currently assigned; Hernick et al., 2008, Flores et al., 2021), most taxa have OBs in all cells (Flores et al., 2018; Romani et al., 2022) (Fig. 5A). This latter pattern has previously been interpreted as the ancestral condition in liverworts (Crandall-Stotler et al., 2005). However, given that the three liverwort classes are estimated to have diverged from a common ancestor no later than the Late Silurian (∼420 Ma), and last shared a common ancestor with mosses in the Late Ordovician (∼450 Ma) (Heinrichs et al., 2007; Bechteler et al., 2023), the occurrence of idioblastic dark cells in *M. sharonae* supports the possibility that idioblastic OBs represent the ancestral state from which other OB cell patterns subsequently evolved.

**Fig. 5.**
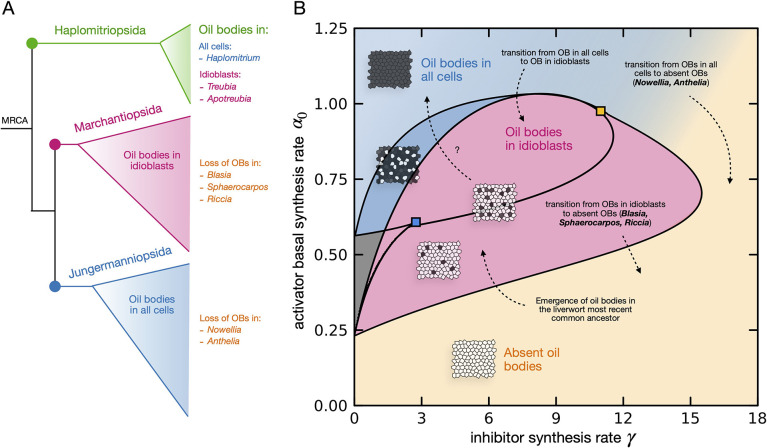
**OB cell evolution through the lens of a flexible patterning module.** (A) OB cell distribution across the liverwort phylogeny. Branch colors indicate the general spatial arrangement of OB cells in the corresponding lineages: Haplomitriopsida (green), Marchantiopsida (magenta) and Jungermanniopsida (blue), together with a description of how OB cells are distributed. MRCA stands for most recent common ancestor. (B) Patterning regimes (Fig. 3C) showing where OBs appear in all cells (light blue), in idioblastic cells (magenta), or are absent (yellow). Phenotypic transitions during evolution can be interpreted as excursions in this space. For instance, the transition from having OBs in all cells to their disappearance (as seen in *Nowellia* or *Anthelia*) can be interpreted either as a smooth change (e.g. through slowly changing environments) or as a sharp transition (e.g. through strong mutations). Similarly, the transition from OBs in idioblasts to OB loss (as occurs in many genera of Marchantiopsida) can be interpreted as the crossing of a bifurcation boundary.

The variation in type and spatial organization of OBs suggests a developmental flexibility that can be co-opted to produce more specialized adaptations suited to changing ecological contexts. Notably, most of the phenotypic variation in OB cell patterns was already present in *M. sharonae*, suggesting that developmental plasticity could have supported timely responses to the changing environments and emerging biotic stresses of Devonian landscapes. In our modeling framework, phenotypic variation reflects the mechanisms' ability to explore different regions of the patterning space (Figs 3C and 5B). Modulating the relative effect of the activator and inhibitor constitutes a route for evolutionary change by shifting the system between different stable states (Alberch, 1991; Jaeger and Monk, 2014). These adjustments can lead to bifurcations in developmental dynamics, in which small variations in parameters can result in large phenotypic changes. In this regard, phenotypic transitions, such as the loss of OBs or the shift from ubiquitous to idioblastic OB distribution, reflect incursions into qualitatively distinct patterning domains (Fig. 5B), which can result from, for example, genetic mutations, biotic stresses, or environmental perturbations. For instance, the presence of OBs in all cells in most species of Jungermanniopsida does not necessarily imply the absence of lateral inhibition (in principle, the inhibitory signal *v* may still diffuse between adjacent cells and repress the activator *u*, but not strongly enough to produce a pattern-forming instability). Different lineages may vary in the expression of particular genes – potentially through gene loss, duplication, or regulatory divergence – which could affect the distribution of OB cells. However, these genes operate within a broader gene regulatory network responsible for pattern formation. Consequently, evolutionary changes may shift the system into a parameter regime in which the underlying logic of lateral inhibition is retained (provided that the core inhibitory components themselves have not been lost), yet regular spatial patterns are no longer stable, resulting instead in homogeneous cellular states. This rationale leads us to hypothesize that the patterning mechanism underlying OB cell specification arose early in liverwort evolution and has persisted despite extensive diversification, being repurposed over time due to lineage-specific selective pressures.

Despite being one of the defining synapomorphies of the group, OBs have rarely been used to explore evolutionary relationships within the liverwort clade, except in a few studies (Schuster, 1966; Crandall-Stotler et al., 2009). Because the spatial organization of OB cells reflects aspects of the underlying developmental system, they may provide useful taxonomic information when considered alongside other morphological traits. Although our results suggest that OB cell patterns can change rapidly on relatively short evolutionary timescales, their distribution across taxa may still shed light on the evolution of patterning and its adaptive role in plant defense. Dark cell patterns very similar in structure and distribution to those of *M. sharonae* have also been described in fossils from the early liverworts *Pallaviciniites devonicus* (Hueber, 1961; Tremblay and Mercadal, 2026) and *Treubiites kidstonii* (Walton, 1925; Krassilov and Schuster, 1984), suggesting idioblastic OBs might have been a widespread trait in early liverworts and further supporting their interpretation as the ancestral state in the most recent common ancestor of liverworts. Strikingly, similar dark cells have been described in Paleozoic mosses (Maslova et al., 2012; Ivanov et al., 2018; Ignatov and Maslova, 2021). Because extant mosses do not possess OBs, the interpretation of these cells as homologous to OB cells would imply either a single evolutionary origin – potentially pre-dating the divergence of bryophyte lineages – followed by a secondary loss in mosses, or an independent evolution and subsequent loss in mosses. Given the sparse fossil record of bryophytes, clarifying these alternatives will be essential for properly resolving their evolutionary history and associated character evolution.

Recent studies in *M. polymorpha* are shedding light on the molecular and developmental mechanisms underlying OB cell specification. The transcription factors MpC1HDZ, MpERF13, and MYB02 act as activators of OB cell specification, as loss-of-function mutants lack OB cells altogether (Romani et al., 2020; Kanazawa et al., 2020; Kubo et al., 2018, 2024). Notably, MpC1HDZ and MpERF13 activate the expression of MYB02 (Kubo et al., 2024). The transcription factor MpTGA represses the expression of both MpC1HDZ and MpERF13, and loss-of-function mutants of MpTGA show a dramatic increase in OB cells. Our model can qualitatively reproduce all these phenotypes (Fig. S10). In addition to modulating the spatial distribution of OB cells, several of these genes are necessary for the synthesis of OB-specific compounds. TGA represses MpSYP12B, an OB-resident SNARE protein that localizes in the OB membrane (Kanazawa et al., 2020; Gutsche et al., 2024), and upregulates terpenoid biosynthesis enzymes encoded by TPS and MTPSL (Gutsche et al., 2024). The transporter MpABCG1 was recently shown to mediate the OB accumulation of sesquiterpenes, compounds that contribute to herbivore resistance (Forestier et al., 2026). Finally, the immune regulator MpNPR has been shown to control the expression of MpSYP12B, MpMYB02, and MpABCG1 through its interaction with MpERF13 (Espinosa-Cores et al., 2026), thereby linking the defensive function of OBs to the developmental processes that underlie OB cell distribution. While these studies are beginning to reveal the core components of the underlying gene regulatory network (Fig. S11), no feedback mechanism capable of cell-type patterning has yet been identified. Nevertheless, similar mechanisms involving local activation and lateral inhibition are known to govern the differentiation and spatial distribution of diverse plant cell types. For instance, the specification of both trichomes and trichoblasts in *Arabidopsis thaliana* involves lateral inhibition processes that regulate the spacing of differentiated cells (Schellmann et al., 2002; Lee and Schiefelbein, 2002). Similarly, in *M. polymorpha*, a mechanism combining local activation and lateral inhibition has recently been shown to govern rhizoid specification (Thamm et al., 2020; Mercadal et al., 2026), demonstrating that such patterning processes operate in liverworts. Given these precedents, it is plausible that OB cell differentiation relies on similar mechanisms. Future characterization of the regulatory network underlying OB cell differentiation will clarify whether OB cells represent yet another example of a widely recurring developmental strategy for generating regularly spaced cell types in plants. Such findings will provide a foundation for comparative analyses of OB cell patterning across liverwort taxa, allowing us to test how the underlying developmental mechanisms have been conserved, modified, or diversified during liverwort evolution.

## MATERIALS AND METHODS

### Sample collection and processing

#### M. sharonae

*M. sharonae* material originates from the Cairo quarry, New York State, USA, located south of New York State Route 145 at the Cairo Highway Department headquarters, 3.22 km NW of Cairo, Greene County, New York (42.32°N and 74.04°W, NAD 83). The material was collected from three locations approximately 3 m apart along a 25 m-wide exposed lens of dark gray shales and mudstones. Shale fragments were immersed in 48% hydrofluoric acid to dissolve the shale matrix. The isolated carbonaceous fossils were rinsed in water until a neutral pH was obtained, and then they were rinsed again twice. Thousands of carbonaceous *M. sharonae* fossils, mostly fragmentary, were isolated. Selected specimens were dehydrated to a 50% glycerine solution, then the fossil fragments were mounted on glass slides and fixed with a permanent slide mounting medium (Glycerin Gelatin). Over 600 permanent slides were created. Samples were photographed with a Nikon D90 digital camera mounted on a Leica DMRB compound microscope. High-resolution images of the mounted samples were acquired with a Leica DM2500 microscope equipped with differential interference contrast, a Plan Apo 63× Oil objective, and a Nikon DS-Fi1 Digital Camera. Extended depth-of-field images were generated using the Adobe Photoshop stacking feature. All fossils are housed in the University of California Museum of Paleontology at the University of California, Berkeley, CA, USA. Accession numbers for the specimens used in this paper are: UC250157.01-UC250157.06, UC250156.01-UC250156.23, UC250154.01, UC250155.01-UC250155.29, UC250153.01-UC250153.10, UC250151.01-UC250151.18, UC250152.01-UC250152.05, UC250150.01-UC250150.18 and UC250149.01-UC250149.10. The full-size specimen in Fig. 1A is shown floating in water and could not be preserved to become a permanent slide.

#### T. lacunosa

Fresh material of *T. lacunosa* (Colenso) Prosk. was collected from four localities in Arthur's Pass National Park, in New Zealand's South Island, and one locality on Stewart Island, under a group permit during the 2012 John Child Workshop (permit number 35273-FLO). Some of the South Island collecting was done through Landcare Research and some under an individual permit. While not endangered, *T. lacunosa* is relatively rare. Because they grow in isolated colonies, collection was limited. A few individual plants were collected from several areas at each locality. Two to six ‘leaf’ tips per plant were used, from the ‘middle’ leaves only from each plant, mainly the unistratose region. Only undamaged samples were used for the analyses. Samples were imaged while the material was still fresh at 100× magnification using a Leica DMRB compound microscope fitted with a Nikon D90 digital camera. Voucher specimens of *T. lacunosa* have been deposited in the University Herbarium at University of California, Berkeley, CA, USA (accession numbers UC2036933, UC2036941, UC2036942, UC2036945 and UC2036946).

#### A. nana

Field work on *A. nana* was primarily conducted in Glacier Bay National Park, AK, USA, under Research Permit GLBA-2023-SCI-0008. The surveys were intuitively controlled and specifically targeted two distinct habitats along the park's outermost coast. *A. nana* was found on partially shaded, liverwort-dominated, humic soil in the forested edges of low-elevation, peat-dominated muskegs, particularly those with a yellow cedar (*Callitropsis nootkatensis*) component. It could also be found on partially shaded, stable, and perennially damp and steeply inclined banks of incised creeks. We used LIDAR data on a GPS device in the field to help locate suitable terrain features. Samples were minimally air-dried before examination under the microscope. A ‘leaf’ was removed from one or two plants from each collection, and a healthy and mostly unistratose apical or lateral margin was removed and mounted on a microscope slide. Photomicroscopy images were captured using a Canon R DSLR, with five to ten images manually stacked and then combined into a single stack with Adobe Photoshop. The collections will be housed at UAAH (University of Alaska, Anchorage Herbarium) and will be available in Bryophyte Portal from November 2026.

### Processing and microscopy of *M. polymorpha*

Tak-1 plants were grown in Jiffy-7 pots for 3-4 weeks under long-day conditions (16 h/8 h) at 22°C and 5000 lux. Thalli were stained with BODIPY 493/503 (Invitrogen, LOT3079935) for 10 min (Kanazawa et al., 2020; Romani et al., 2020). A stock solution of 1 mg/ml in dimethyl sulfoxide (DMSO) was diluted to a 1 μg/ml solution in PBS. Afterwards, the plants were washed for 1 min in PBS. For images obtained with both propidium iodide (PI) and BODIPY staining, plant samples were first incubated with PI (10 μg/ml) for 20 min. Afterward, the samples were washed in PBS for 1 min before being stained with BODIPY as described above. Imaging was performed using a confocal laser scanning microscope (Leica SP8) with the following settings: BODIPY (excitation: 488 nm; emission: 520-550 nm); PI (excitation: 550 nm; emission: 620-650 nm). For imaging PI and BODIPY, lasers were used in a sequential scan.

### Analysis of spatial statistics

To analyze the statistical properties of dark/OB cell patterns, we performed cell segmentation of thalli sections (Fig. S1A) with the deep learning-based cell segmentation software cellpose (Stringer et al., 2021), using the ‘cyto2’ model with a diameter parameter ranging from 50 to 100 pixels (Fig. S1B). For *M. sharonae*, *T. lacunosa*, and *A. nana*, segmentation was performed directly from the cell walls observed in the images. Segmentation of *M. polymorpha* was performed using cell boundaries seen through BODIPY (Fig. S8). For all species, semi-automatic segmentation was followed by manual correction of cells that were badly segmented. Finally, we classified cell types (dark/OB cells versus light/non-OB cells) with ilastik (Berg et al., 2019) (Fig. S1C). After segmentation and classification, we extracted the cell positions (centroids) from the labeled image. We then used the Python package NetworkX (Hagberg et al., 2008) to construct the cellular network, from which cell connectivity properties (cell neighbors, shortest paths between pairs of cells, etc.) can be extracted (Jackson et al., 2017; Fischer et al., 2023) (Fig. S1D). To compute the distances between neighboring dark/OB cells, we first defined dark/OB cell neighborhoods by constructing a Gabriel graph of the corresponding nodes (Fig. S1E). In a Gabriel graph, an edge between two nodes *i* and *j* exists only if the disk for which the diameter is the line connecting the nodes *i* and *j* contains no other nodes (Gabriel and Sokal, 1969). This approach has previously been used to quantify the network structure of trichome patterns in *Arabidopsis* (Greese et al., 2012) and rhizoids in *Marchantia* (Mercadal et al., 2026). Once the pairwise connections between dark/OB cells have been established, we computed the shortest paths between pairs of neighboring dark/OB cells using the ‘shortest_path’ function of NetworkX (Hagberg et al., 2008) (Fig. S1F). This provided all the necessary information to compute the cluster sizes, Moran's *I*, and the distribution of dark/OB cell distances. We used the same pipeline to analyze dark/OB cell patterns in all the model simulations.

To quantify the spatial distribution of dark/OB cell patterns, we used the following metrics associated with each sample/simulation:

- Dark/OB cell frequency: The fraction of dark/OB cells over the total number of cells in that sample/simulation.
- Frequency distribution of *k*-clusters, *f*_*k*_: The number of *k*-clusters of dark/OB cells divided by the total number of clusters of that sample/simulation.
- Idioblast proportion: The proportion of dark/OB cell clusters of size *k* = 1, i.e. from the total number of clusters, the percentage of those with a single dark/OB cell.
- Moran's *I* statistic: A measure of spatial autocorrelation defined as:

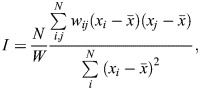

where *N* is the total number of cells in the sample/simulation, *x*_*i*_ is the value associated with each cell (*x*_*i*_ = 1 for dark/OB cells, *x*_*i*_ = 0 for epidermal cells), 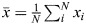 is the average value of *x*_*i*_ (equivalent to the frequency of dark/OB cells), *w*_*ij*_ is the spatial weight between cells *i* and *j*, and 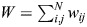 is the sum of all spatial weights, acting as a normalizing factor. For the weights *w*_*ij*_, we used a binary adjacency matrix such that *w*_*ij*_ = 1 if cells *i* and *j* are neighbors and *w*_*ij*_ = 0 otherwise. This choice reflects the assumption that spatial interactions occur between adjacent cells, consistent with the discrete cellular structure of the model. *I* > 0 indicates positive spatial autocorrelation or clustering, whereas *I* < 0 indicates negative spatial autocorrelation or dispersion. *I* ≈ 0 indicates randomness, without regularity in the spatial pattern.

- Distance frequency, *D*_*j*_: The Gabriel graph defines a dark/OB cell neighborhood where each pair of cells is separated by *j* epidermal cells (Fig. S1E,F). This separation is computed as the shortest path between pairs of dark/OB cells, i.e. the minimum number of epidermal cells connecting pairs of dark/OB cells (Fig. S1F). *D*_*j*_ is defined as the frequency of these shortest paths. With this definition, dark/OB cells separated by *j* = 0 epidermal cells are adjacent and belong to the same cluster.
- Average dark/OB cell distance 〈*λ*〉 and coefficient of variation 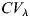: 〈*λ*〉 denotes the mean value of the distance frequency, 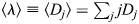. If 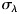 is its associated standard deviation 
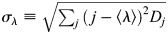
, the coefficient of variation is defined as 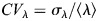.

For consistency, we performed the statistical analysis of *T. lacunosa*, *A. nana*, and *M. polymorpha* using sections with a similar number of cells as in *M. sharonae*.

### Model simulation and analysis

We simulated all models in irregular cell lattices that mimic the tissue layouts of mature thalli. These lattices were generated by considering a hexagonal array of cells in the presence of spatial noise. To do this, we created two-dimensional points arranged in a perfect triangular lattice with positions 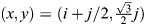 where *i*, *j* are integers. We then randomly displaced each point a certain distance from its original location, i.e. 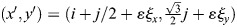, where (*ξ*_*x*_, *ξ*_*y*_) are random numbers taken from a uniform distribution between 0 and 1, and ε sets the variability from the perfect hexagonal layout. If ε is small, the lattice is more or less hexagonal, with mostly six-sided cells, whereas for large values, four- and three-sided cells can appear. After choosing a value for the irregularity that mimics the heterogeneity of cellular tissues (ε = 0.25), a Voronoi tessellation was created from the resulting points, generating the cellular layouts used in our simulations.

### Simulation of random patterns

To generate random patterns of dark/OB cells, every cell in a tissue of size *N*_*t*_ = *N*_*x*_ × *N*_*y*_ performs a binary decision to become a dark/OB cell with probability *p* or an epidermal cell with probability *q* = 1 − *p*. The spatial statistics in this random model are completely determined by the parameters *p* and *N*_*t*_. In particular, the total number of dark/OB cells is a random variable *X* that follows a binomial distribution with mean 〈*X*〉 = *N*_*t*_*p* and standard deviation 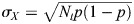. The average frequency of dark/OB cells is 
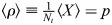
.

### Model with local activation and lateral inhibition

We modeled dark/OB cell patterning using a discrete reaction-diffusion system with local activation and lateral inhibition, allowing for cell-autonomous bistability, and with diffusion restricted to the inhibitor. The model is similar to one used to describe rhizoid precursor patterning in *M. polymorpha* (Mercadal et al., 2026). Each cell in our model is labeled with an index *i*∈[1,…, *N*_*t*_] and is characterized by its concentrations of *u*_*i*_ (activator) and *v*_*i*_ (inhibitor), which dynamically change over time according to the following processes. We assume the activator has a basal synthesis rate *α*_0_ and can self-activate with maximum rate *α*, activation threshold *k*_*u*_, and cooperativity exponent *n*_*u*_. Degradation of *u* occurs both linearly at rate *d*_*u*_ and via *v* at rate *k*_*c*_. The inhibitor *v* is produced at a basal rate *γ*_0_ and is activated by *u* with a maximum rate *γ*, an activation threshold *k*_*v*_, and a cooperativity exponent *n*_*v*_. Finally, *v* can be linearly degraded at rate *d*_*v*_ and can move from cell to cell with a diffusion coefficient *D*_*v*_. Taken together, these processes are modeled through the following set of coupled ordinary differential equations for the concentrations in each cell:

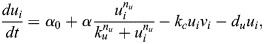


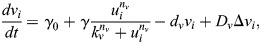
where Δ is a discrete Laplacian operator for an irregular lattice, defined as:

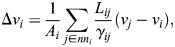
where *A*_*i*_ is the area of cell *i*, *L*_*ij*_ is the length of the shared edges between cells *i* and *j*, *γ*_*ij*_ the distance between centroids of cells *i* and *j*, and *nn*_*i*_ the nearest neighbors of cell *i* (Sukumar and Bolander, 2003). In the model, lateral inhibition is driven by the diffusive transport of *v* to adjacent cells and the subsequent degradation of *u* molecules. To simulate the patterns, we solved the system of coupled differential equations using the Runge-Kutta 4 method with a time step *dt* = 0.02 until the patterns became stationary 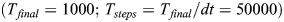. Boundary conditions are such that cells in the tissue boundaries only interact with their adjacent neighbors. In Table S1, we show the parameter values used for the simulations. We defined dark/OB cells as those with steady-state levels of *u* equal to or higher than the threshold value *u*_*T*_ = 1. We used *γ* and *α*_0_ as control parameters to analyze the stability and variability of dark/OB cell patterns (Fig. 3C; Figs S4, S5).

### Initial conditions

To initialize our model simulations, we considered uniformly distributed random numbers of the form 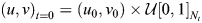, where *N*_*t*_ denotes the number of cells in the tissue. We chose *u*_0_ = 1.35 and *v*_0_ = 0.4 for all our simulations.

### Parameter exploration

To identify the parameters that reproduce the spatial statistics of dark/OB cell patterns, we performed simulations on cellular tissues of size 25×20 and sampled a 100×100 grid of (*γ*, *α*_0_) values, with *γ* ∈ [0, 18] and *α*_0_ ∈ [0, 1.25], giving 10,000 parameter combinations. The remaining parameters were fixed (Table S1). For each category, we identified the pairs (*γ*, *α*_0_) that generated patterns in which all metrics (dark/OB cell frequency, idioblast proportion, Moran's *I*, 〈*λ*〉, 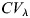) simultaneously lay within the experimental ranges. These pairs are defined by the intersection (denoted by the symbol 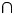) of admissible regions for each metric, i.e. the set 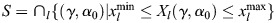, where *X*_*l*_(*γ*, *α*_0_) denotes the value of metric *l*, and 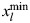 and 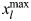 represent, respectively, the minimum and maximum values of each experimental metric (Figs S6 and S7). This set constitutes a 5-dimensional hypercube that encloses all sample points. Since strictly adhering to the bounds 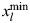, 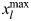 may result in a small number of simulations that align with the experimental patterns, in some cases we introduce small tolerance parameters *δ*_−_ and *δ*_+_, associated with 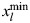 and 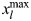, respectively. This adjustment expands the acceptable range, allowing it to span from 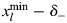 to 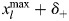. In Figs S6 and S7, we show these bounds for each metric as gray regions. In Table S2, we show 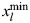, 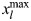, *δ*_−_, and *δ*_+_ associated with each metric for each pattern type in *M. sharonae*, and for the extant taxa.

### Bifurcations and linear stability analysis

We performed linear stability analysis (Strogatz, 2018; Collier et al., 1996) of our model to find the parameter space regions where patterns emerge spontaneously from a homogeneous state due to small perturbations (Fig. 3C, region 6). For this analysis, we considered a hexagonal lattice of size 25×20, where the spatial position of each cell is defined by the indices *j*, *k*. To find the stability of a homogeneous state, we first numerically compute the stationary homogeneous states by computing the fixed points of the uncoupled system. We denote a stationary homogeneous solution as the vector 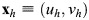. Next, we consider a perturbation of the form 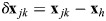, where 
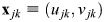
 is a vector of all the system's variables. We assume perturbations of the form:

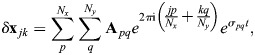
where *p*, *q* denote the Fourier modes, i the imaginary number, 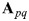 is the modes' amplitude, and *σ*_*pq*_ is their growth rate. The dynamics of these perturbations are analyzed in the linearized version of the model around the homogeneous state. Therefore, the growth rates *σ*_*pq*_ correspond to the eigenvalues of the Jacobian matrix:

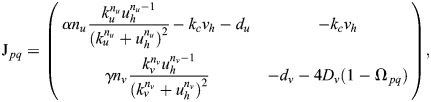
where 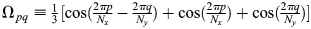. This quantity ranges from 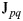, having its minimum when all terms in the brackets equal 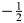, and its maximum when they equal to 1. The corresponding eigenvalue equation takes the form of a rank-2 polynomial *λ*^2^ + *Aλ* + *B* = 0, where the coefficients *A*, *B* are constants associated with the Jacobian 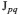 evaluated at the homogeneous states. The homogeneous state is linearly unstable when the real part of any of the growth rates *σ*_*pq*_ is positive (Re(*σ*_*pq*_) > 0). We solved the eigenvalue equation numerically for different values of the parameters (*γ*, *α*_0_) to find the parameter values for which the condition of instability (*Re*(*σ*_*pq*_) > 0) is met for any (*p*, *q*). The spatial modes that first become unstable (those with 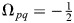) were *p* = *N*_*x*_/3 and *q* = 2*N*_*y*_/3 (and their reciprocals *p* = 2*N*_*x*_/3 and *q* = *N*_*y*_/3), i.e. salt-and-pepper patterns. In Fig. S4B, we depict the Turing bifurcations [Re(*λ*) = 0 for inhomogeneous modes, Re(*λ*) < 0 for homogeneous modes; dark pink curves] and the regions of spontaneous pattern formation [Re(*λ*) > 0 for inhomogeneous modes, Re(*λ*) < 0 for homogeneous modes; light and dark pink regions] in the plane (*γ*, *α*_0_). An equivalent linear stability analysis was performed to obtain region 6 in Fig. S5. To compute the bifurcations in Fig. 3C, Fig. S4, and Fig. S5, we used custom-made software and the Julia package BifurcationKit (https://hal.archives-ouvertes.fr/hal-02902346).

### Patterning regimes of the system

In Fig. 3C, we show all the possible patterning regimes in the (*γ*, *α*_0_) plane. If *α*_0_ (production of the activator) is small, dark cells do not appear (region 1). Conversely, if *α*_0_ is large, all cells become dark. For intermediate values of *α*_0_ and *γ*, patterns can occur. In Fig. S4, we show all these patterning regimes and their associated bifurcations and instabilities. Without diffusion (*D*_*v*_ = 0), the system is equivalent to a single-cell system and exhibits a region of cell-autonomous bistability, bounded by saddle-node bifurcations (Fig. S4A, gray region). These states correspond to homogeneous states in the spatially extended system. In the bistable region, both homogeneous states (no dark cells and all dark cells) are linearly stable, and the emergence of dark cell patterns depends exclusively on the initial conditions. When diffusion is introduced, diverse patterning regimes emerge (Fig. S4B). Linear stability analysis reveals a region of spontaneous pattern formation enclosed by subcritical Turing bifurcations (Fig. S4B, pink region). Here, patterns emerge spontaneously, span the whole tissue, and tend to display spatial regularity (Fig. S4D). A small part of this region co-exists with the low-*u* homogeneous stable state (dark pink region). Here, high-*u* states can lose stability due to small inhomogeneous perturbations, whereas the low-*u* homogeneous state is linearly stable. In the gray region, both homogeneous states (low-*u* and high-*u*) are linearly stable, and coincide with the same region in the cell-autonomous case. Outside regions of spontaneous pattern formation, regular patterns can still emerge (localized states I, II; turquoise regions). In these regions, pattern solutions co-exist with homogeneous states, are nonlinear, and depend on initial conditions. Localized patterning regions are enclosed by saddle-node bifurcations (dashed turquoise curves in Fig. S4B,C) corresponding to the pattern states that first become stable, i.e. the state with all dark cells and a single light cell (localized patterns I), and the state with a single dark cell in a tissue of light cells (localized patterns II).

## Supplementary Material



10.1242/develop.205696_sup1 Supplementary information

## References

[DEV205696C1] Alberch, P. (1991). From genes to phenotype: dynamical systems and evolvability. *Genetica* 84, 5-11. 10.1007/BF00123979 1874440

[DEV205696C2] Amundson, R. (2005). *The Changing Role of the Embryo in Evolutionary Thought: Roots of Evo-Devo*. Cambridge University Press.

[DEV205696C3] Bechteler, J., Peñaloza-Bojacá, G., Bell, D., Gordon Burleigh, J., McDaniel, S. F., Christine Davis, E., Sessa, E. B., Bippus, A., Christine Cargill, D., Chantanoarrapint, S. et al. (2023). Comprehensive phylogenomic time tree of bryophytes reveals deep relationships and uncovers gene incongruences in the last 500 million years of diversification. *Am. J. Bot.* 110, e16249. 10.1002/ajb2.16249 37792319

[DEV205696C4] Berg, S., Kutra, D., Kroeger, T., Straehle, C. N., Kausler, B. X., Haubold, C., Schiegg, M., Ales, J., Beier, T., Rudy, M. et al. (2019). Ilastik: interactive machine learning for (bio) image analysis. *Nat. Methods* 16, 1226-1232. 10.1038/s41592-019-0582-9 31570887

[DEV205696C5] Boyce, C. K. (2008). The fossil record of plant physiology and development – what leaves can tell us. *Paleontol. Soc. Papers* 14, 133-146. 10.1017/S1089332600001650

[DEV205696C6] Chipman, A. D. and Edgecombe, G. D. (2019). Developing an integrated understanding of the evolution of arthropod segmentation using fossils and evo-devo. *Proc. R. Soc. B* 286, 20191881. 10.1098/rspb.2019.1881 PMC6790758 31575373

[DEV205696C7] Cohen, M., Georgiou, M., Stevenson, N. L., Miodownik, M. and Baum, B. (2010). Dynamic filopodia transmit intermittent delta-notch signaling to drive pattern refinement during lateral inhibition. *Dev. Cell* 19, 78-89. 10.1016/j.devcel.2010.06.006 20643352

[DEV205696C8] Collier, J. R., Monk, N. A., Maini, P. K. and Lewis, J. H. (1996). Pattern formation by lateral inhibition with feedback: a mathematical model of delta-notch intercellular signalling. *J. Theor. Biol.* 183, 429-446. 10.1006/jtbi.1996.0233 9015458

[DEV205696C9] Cooper, R. L., Thiery, A. P., Fletcher, A. G., Delbarre, D. J., Rasch, L. J. and Fraser, G. J. (2018). An ancient turing-like patterning mechanism regulates skin denticle development in sharks. *Sci. Adv.* 4, eaau5484. 10.1126/sciadv.aau5484 30417097 PMC6221541

[DEV205696C10] Crandall-Stotler, B. J., Forrest, L. L. and Stotler, R. E. (2005). Evolutionary trends in the simple thalloid liverworts (Marchantiophyta, Jungermanniopsida subclass Metzgeriidae). *Taxon* 54, 299-316. 10.2307/25065462

[DEV205696C11] Crandall-Stotler, B., Stotler, R. and Long, D. (2009). Phylogeny and classification of the Marchantiophyta. *Edinb. J. Bot.* 66, 155-198. 10.1017/S0960428609005393

[DEV205696C12] Cunningham, J. A., Rahman, I. A., Lautenschlager, S., Rayfield, E. J. and Donoghue, P. C. (2014). A virtual world of paleontology. *Trends Ecol. Evol.* 29, 347-357. 10.1016/j.tree.2014.04.004 24821516

[DEV205696C13] Delaux, P.-M., Hetherington, A. J., Coudert, Y., Delwiche, C., Dunand, C., Gould, S., Kenrick, P., Li, F.-W., Philippe, H., Rensing, S. A. et al. (2019). Reconstructing trait evolution in plant evo-devo studies. *Curr. Biol.* 29, R1110-R1118. 10.1016/j.cub.2019.09.044 31689391

[DEV205696C14] DiFrisco, J. and Jaeger, J. (2021). Homology of process: developmental dynamics in comparative biology. *Interface Focus* 11, 20210007. 10.1098/rsfs.2021.0007 34055306 PMC8086918

[DEV205696C84] Espinosa-Cores, L., Michavila, S., Gonzalez-Zuloaga, M., Izquierdo, Y., Solano, R. and Gimenez-Ibanez, S. (2026). Modulation of lineage-specific oil body development and defense against gastropod herbivory in Marchantia polymorpha by MpNPR. *Curr. Biol*. 36, 3271-3283.e5. 10.1016/j.cub.2026.05.053 42320466

[DEV205696C15] Fischer, S. C., Bassel, G. W. and Kollmannsberger, P. (2023). Tissues as networks of cells: towards generative rules of complex organ development. *J. R. Soc. Interface* 20, 20230115. 10.1098/rsif.2023.0115 37491909 PMC10369035

[DEV205696C16] Flores, J. R., Catalano, S. A., Muñoz, J. and Suárez, G. M. (2018). Combined phylogenetic analysis of the subclass Marchantiidae (Marchantiophyta): towards a robustly diagnosed classification. *Cladistics* 34, 517-541. 10.1111/cla.12225 34706484

[DEV205696C17] Flores, J. R., Bippus, A. C., Suárez, G. M. and Hyvönen, J. (2021). Defying death: incorporating fossils into the phylogeny of the complex thalloid liverworts (Marchantiidae, Marchantiophyta) confirms high order clades but reveals discrepancies in family-level relationships. *Cladistics* 37, 231-247. 10.1111/cla.12442 34478198

[DEV205696C18] Forestier, E. C., Asprilla, P., Bonter, I., Romani, F., Frangedakis, E. and Haseloff, J. (2026). Spatial distribution of isoprenoid enzymes and MpABCG1 transporter influences sesquiterpene accumulation in Marchantia polymorpha oil bodies. *Commun. Biol.* 9, 521. 10.1038/s42003-025-09508-4 41772152 PMC13068942

[DEV205696C19] Gabriel, K. R. and Sokal, R. R. (1969). A new statistical approach to geographic variation analysis. *Syst. Zool.* 18, 259-278. 10.2307/2412323

[DEV205696C20] Gilbert, S. F. (2003). The morphogenesis of evolutionary developmental biology. *Int. J. Dev. Biol.* 47, 467. 10.1387/ijdb.14756322 14756322

[DEV205696C21] Greese, B., Wester, K., Bensch, R., Ronneberger, O., Timmer, J., Hulskamp, M. and Fleck, C. (2012). Influence of cell-to-cell variability on spatial pattern formation. *IET Syst. Biol.* 6, 143-153. 10.1049/iet-syb.2011.0050 23039695

[DEV205696C22] Guisoni, N., Martinez-Corral, R., Garcia-Ojalvo, J. and de Navascués, J. (2017). Diversity of fate outcomes in cell pairs under lateral inhibition. *Development* 144, 1177-1186. 10.1242/dev.137950 28174242

[DEV205696C23] Gutsche, N., Koczula, J., Trupp, M., Holtmannspötter, M., Appelfeller, M., Rupp, O., Busch, A. and Zachgo, S. (2024). Mp tga, together with mp npr, regulates sexual reproduction and independently affects oil body formation in Marchantia polymorpha. *New Phytol.* 241, 1559-1573. 10.1111/nph.19472 38095258

[DEV205696C24] Hagberg, A., Swart, P. J. and Schult, D. A. (2008). Exploring network structure, dynamics, and function using NetworkX (No. LA-UR-08-05495; LA-UR-08-5495). Los Alamos National Laboratory (LANL). 10.25080/TCWV9851

[DEV205696C25] Hall, B. K. (2002). Palaeontology and evolutionary developmental biology: a science of the nineteenth and twenty–first centuries. *Palaeontology* 45, 647-669. 10.1111/1475-4983.00253

[DEV205696C26] Harjunmaa, E., Seidel, K., Häkkinen, T., Renvoisé, E., Corfe, I. J., Kallonen, A., Zhang, Z.-Q., Evans, A. R., Mikkola, M. L., Salazar-Ciudad, I. et al. (2014). Replaying evolutionary transitions from the dental fossil record. *Nature* 512, 44-48. 10.1038/nature13613 25079326 PMC4252015

[DEV205696C27] Haug, C. and Haug, J. T. (2021). Methods and practices in paleo-evo-devo. In *Evolutionary Developmental Biology: A Reference Guide* (ed. L. N. de la Rosa and G.B. Müller), pp. 1151-1164.

[DEV205696C28] He, X., Sun, Y. and Zhu, R.-L. (2013). The oil bodies of liverworts: unique and important organelles in land plants. *Crit. Rev. Plant Sci.* 32, 293-302. 10.1080/07352689.2013.765765

[DEV205696C29] Heinrichs, J., Hentschel, J., Wilson, R., Feldberg, K. and Schneider, H. (2007). Evolution of leafy liverworts (Jungermanniidae, Marchantiophyta): estimating divergence times from chloroplast DNA sequences using penalized likelihood with integrated fossil evidence. *Taxon* 56, 31-44. https://www.jstor.org/stable/25065733

[DEV205696C30] Hernick, L. V., Landing, E. and Bartowski, K. E. (2008). Earth's oldest liverworts – Metzgeriothallus sharonae sp. nov. from the middle Devonian (Givetian) of eastern New York, USA. *Rev. Palaeobot. Palynol.* 148, 154-162. 10.1016/j.revpalbo.2007.09.002

[DEV205696C31] Hetherington, A. J. (2024). The role of fossils for reconstructing the evolution of plant development. *Development* 151, dev204322. 10.1242/dev.204322 39417682 PMC11529274

[DEV205696C32] Hetherington, A. J., Dubrovsky, J. G. and Dolan, L. (2016). Unique cellular organization in the oldest root meristem. *Curr. Biol.* 26, 1629-1633. 10.1016/j.cub.2016.04.072 27265396 PMC4920953

[DEV205696C33] Hiscock, T. W. and Megason, S. G. (2015). Mathematically guided approaches to distinguish models of periodic patterning. *Development* 142, 409-419. 10.1242/dev.107441 25605777 PMC4302999

[DEV205696C34] Hueber, F. M. (1961). Hepaticites devonicus: a new fossil liverwort from the Devonian of New York. *Ann. Mo. Bot. Gard.* 48, 125-131. 10.2307/2394879

[DEV205696C35] Ignatov, M. S. and Maslova, E. V. (2021). Fossil mosses: what do they tell us about moss evolution? *Bryophyte Divers. Evol.* 43, 72-97. 10.11646/bde.43.1.7

[DEV205696C36] Ivanov, O. V., Maslova, E. V. and Ignatov, M. S. (2018). Development of the sphagnoid areolation pattern in leaves of palaeozoic protosphagnalean mosses. *Ann. Bot.* 122, 915-925. 10.1093/aob/mcy046 29659704 PMC6215045

[DEV205696C37] Jackson, M. D., Duran-Nebreda, S. and Bassel, G. W. (2017). Network-based approaches to quantify multicellular development. *J. R. Soc. Interface* 14, 20170484. 10.1098/rsif.2017.0484 29021161 PMC5665831

[DEV205696C38] Jaeger, J. and Monk, N. (2014). Bioattractors: dynamical systems theory and the evolution of regulatory processes. *J. Physiol.* 592, 2267-2281. 10.1113/jphysiol.2014.272385 24882812 PMC4048087

[DEV205696C39] Kanazawa, T., Morinaka, H., Ebine, K., Shimada, T. L., Ishida, S., Minamino, N., Yamaguchi, K., Shigenobu, S., Kohchi, T., Nakano, A. et al. (2020). The liverwort oil body is formed by redirection of the secretory pathway. *Nat. Commun.* 11, 6152. 10.1038/s41467-020-19978-1 33262353 PMC7708844

[DEV205696C40] Kjær, K. H., Winther Pedersen, M., De Sanctis, B., De Cahsan, B., Korneliussen, T. S., Michelsen, C. S., Sand, K. K., Jelavić, S., Ruter, A. H., Schmidt, A. M. et al. (2022). A 2-million-year-old ecosystem in Greenland uncovered by environmental dna. *Nature* 612, 283-291. 10.1038/s41586-022-05453-y 36477129 PMC9729109

[DEV205696C41] Krassilov, V. and Schuster, R. (1984). Paleozoic and mesozoic fossils. In *New Manual of Bryology* (ed. R. M. Schuster), vol. 2, pp. 1172-1193. Hattori Botanical Laboratory.

[DEV205696C42] Kubo, H., Nozawa, S., Hiwatashi, T., Kondou, Y., Nakabayashi, R., Mori, T., Saito, K., Takanashi, K., Kohchi, T. and Ishizaki, K. (2018). Biosynthesis of riccionidins and marchantins is regulated by R2R3-MYB transcription factors in Marchantia polymorpha. *J. Plant Res.* 131, 849-864. 10.1007/s10265-018-1044-7 29845372

[DEV205696C43] Kubo, H., Sunhwa, K., Teramori, H. and Takanashi, K. (2024). MpR2R3-MYB2 is a key regulator of oil body formation in Marchantia polymorpha. *Planta* 260, 68. 10.1007/s00425-024-04498-9 39120651

[DEV205696C44] Labandeira, C. C., Tremblay, S. L., Bartowski, K. E. and VanAller Hernick, L. (2014). Middle Devonian liverwort herbivory and antiherbivore defence. *New Phytol.* 202, 247-258. 10.1111/nph.12643 24372344

[DEV205696C45] Lee, M. M. and Schiefelbein, J. (2002). Cell pattern in the Arabidopsis root epidermis determined by lateral inhibition with feedback. *Plant Cell* 14, 611-618. 10.1105/tpc.010434 11910008 PMC150583

[DEV205696C46] Maslova, E. V. M., Mosseichik, Y. V., Ignatiev, I. A., Ivanov, O. V. and Ignatov, M. S. (2012). On the leaf development in palaeozoic mosses of the order Protosphagnales. *Arctoa* 21, 241-264. 10.15298/arctoa.21.24

[DEV205696C47] Masterson, J. (1994). Stomatal size in fossil plants: evidence for polyploidy in majority of angiosperms. *Science* 264, 421-424. 10.1126/science.264.5157.421 17836906

[DEV205696C48] McElwain, J. C. and Steinthorsdottir, M. (2017). Paleoecology, ploidy, paleoatmospheric composition, and developmental biology: a review of the multiple uses of fossil stomata. *Plant Physiol.* 174, 650-664. 10.1104/pp.17.00204 28495890 PMC5462064

[DEV205696C49] Meinhardt, H. and Gierer, A. (2000). Pattern formation by local self-activation and lateral inhibition. *BioEssays* 22, 753-760. 10.1002/1521-1878(200008)22:8<753::AID-BIES9>3.0.CO;2-Z 10918306

[DEV205696C50] Mercadal, J., Ferreira-Guerra, M., Caño-Delgado, A. I. and Ibañes, M. (2026). Cell-type specification at criticality underlies rhizoid patterning in the liverwort Marchantia polymorpha. *Cell Rep.* 45, 116728. 10.1016/j.celrep.2025.116728 41422508

[DEV205696C51] Onimaru, K., Marcon, L., Musy, M., Tanaka, M. and Sharpe, J. (2016). The fin-to-limb transition as the re-organization of a Turing pattern. *Nat. Commun.* 7, 11582. 10.1038/ncomms11582 27211489 PMC4879262

[DEV205696C52] Ortiz, A., Bailey, S. E., Schwartz, G. T., Hublin, J.-J. and Skinner, M. M. (2018). Evo-devo models of tooth development and the origin of hominoid molar diversity. *Sci. Adv.* 4, eaar2334. 10.1126/sciadv.aar2334 29651459 PMC5895448

[DEV205696C53] Petrone-Mendoza, E., Vergara-Silva, F. and Olson, M. E. (2023). Plant morpho evo-devo. *Trends Plant Sci.* 28, 1257-1276. 10.1016/j.tplants.2023.06.007 37423784

[DEV205696C54] Raspopovic, J., Marcon, L., Russo, L. and Sharpe, J. (2014). Digit patterning is controlled by a Bmp-Sox9-Wnt Turing network modulated by morphogen gradients. *Science* 345, 566-570. 10.1126/science.1252960 25082703

[DEV205696C55] Romani, F., Banić, E., Florent, S. N., Kanazawa, T., Goodger, J. Q., Mentink, R. A., Dierschke, T., Zachgo, S., Ueda, T., Bowman, J. L. et al. (2020). Oil body formation in Marchantia polymorpha is controlled by mpc1hdz and serves as a defense against arthropod herbivores. *Curr. Biol.* 30, 2815-2828. 10.1016/j.cub.2020.05.081 32559445

[DEV205696C56] Romani, F., Flores, J. R., Tolopka, J. I., Suárez, G., He, X. and Moreno, J. E. (2022). Liverwort oil bodies: diversity, biochemistry, and molecular cell biology of the earliest secretory structure of land plants. *J. Exp. Bot.* 73, 4427-4439. 10.1093/jxb/erac134 35394035

[DEV205696C57] Rothwell, G. and Lev-Yadun, S. (2005). Evidence of polar auxin flow in 375 million-year-old fossil wood. *Am. J. Bot.* 92, 903-906. 10.3732/ajb.92.6.903 21652473

[DEV205696C58] Rothwell, G. W. and Tomescu, A. M. (2021). Structural fingerprints of development at the intersection of evo-devo and the fossil record. In *Evolutionary Developmental Biology: a Reference Guide* (ed. L. N. de la Rosa and G.B. Müller), pp. 573-602. Springer.

[DEV205696C59] Rothwell, G. W., Sanders, H., Wyatt, S. E. and Lev-Yadun, S. (2008). A fossil record for growth regulation: the role of auxin in wood evolution1. *Ann. Mo. Bot. Gard.* 95, 121-134. 10.3417/2006208

[DEV205696C60] Rudall, P. J. and Bateman, R. M. (2019). Leaf surface development and the plant fossil record: stomatal patterning in bennettitales. *Biol. Rev.* 94, 1179-1194. 10.1111/brv.12497 30714286

[DEV205696C61] Salazar-Ciudad, I. and Jernvall, J. (2010). A computational model of teeth and the developmental origins of morphological variation. *Nature* 464, 583-586. 10.1038/nature08838 20220757

[DEV205696C62] Salazar-Ciudad, I., Jernvall, J. and Newman, S. A. (2003). Mechanisms of pattern formation in development and evolution. *Development* 130, 2027-2037. 10.1242/dev.00425 12668618

[DEV205696C63] Sanders, H., Rothwell, G. W. and Wyatt, S. (2007). Paleontological context for the developmental mechanisms of evolution. *Int. J. Plant Sci.* 168, 719-728. 10.1086/513519

[DEV205696C64] Schellmann, S., Schnittger, A., Kirik, V., Wada, T., Okada, K., Beermann, A., Thumfahrt, J., Jürgens, G. and Hülskamp, M. (2002). Triptychon and caprice mediate lateral inhibition during trichome and root hair patterning in Arabidopsis. *EMBO J.* 21, 5036-5046. 10.1093/emboj/cdf524 12356720 PMC129046

[DEV205696C65] Schuster, R. M. (1966). *The Hepaticae and Anthocerotae of North America*, Vol. 1-6. New York, NY, USA and London, UK: Columbia University Press; Vol. 5 & 6. Chicago, IL, USA: Field Museum of Natural History.

[DEV205696C66] Schweisguth, F. and Corson, F. (2019). Self-organization in pattern formation. *Dev. Cell* 49, 659-677. 10.1016/j.devcel.2019.05.019 31163171

[DEV205696C67] Schweizer, F., Monte, I., Solano, R. and Reymond, P. (2025). Marchantia polymorpha defense against snail herbivory. *Plant Environ. Interact.* 6, e70052. 10.1002/pei3.70052 40236298 PMC11997372

[DEV205696C68] Sepkoski, D. (2005). Stephen Jay Gould, Jack Sepkoski, and the ‘quantitative revolution’ in American paleobiology. *J. Hist. Biol.* 38, 209-237. 10.1007/s10739-004-2084-5

[DEV205696C69] Staps, M., Miller, P. W., Tarnita, C. E. and Mallarino, R. (2023). Development shapes the evolutionary diversification of rodent stripe patterns. *Proc. Natl Acad. Sci. USA* 120, e2312077120. 10.1073/pnas.2312077120 37871159 PMC10636316

[DEV205696C70] Stewart, T. A., Lemberg, J. B., Taft, N. K., Yoo, I., Daeschler, E. B. and Shubin, N. H. (2020). Fin ray patterns at the fin-to-limb transition. *Proc. Natl Acad. Sci. USA* 117, 1612-1620. 10.1073/pnas.1915983117 31888998 PMC6983361

[DEV205696C71] Stringer, C., Wang, T., Michaelos, M. and Pachitariu, M. (2021). Cellpose: a generalist algorithm for cellular segmentation. *Nat. Methods* 18, 100-106. 10.1038/s41592-020-01018-x 33318659

[DEV205696C72] Strogatz, S. H. (2018). *Nonlinear Dynamics and Chaos: with Applications to Physics, Biology, Chemistry, and Engineering*. CRC press.

[DEV205696C73] Sukumar, N. and Bolander, J. (2003). Numerical computation of discrete differential operators on non-uniform grids. *Comput. Mod. Eng. Sci.* 4, 691-706. 10.3970/cmes.2003.004.691

[DEV205696C74] Summers, H. D., Wills, J. W. and Rees, P. (2022). Spatial statistics is a comprehensive tool for quantifying cell neighbor relationships and biological processes via tissue image analysis. *Cell Rep. Method.* 2, 100348. 10.1016/j.crmeth.2022.100348 PMC9701617 36452868

[DEV205696C75] Tanaka, M., Esaki, T., Kenmoku, H., Koeduka, T., Kiyoyama, Y., Masujima, T., Asakawa, Y. and Matsui, K. (2016). Direct evidence of specific localization of sesquiterpenes and marchantin a in oil body cells of Marchantia polymorpha L. *Phytochemistry* 130, 77-84. 10.1016/j.phytochem.2016.06.008 27406893

[DEV205696C76] Thamm, A., Saunders, T. E. and Dolan, L. (2020). MpFEW RHIZOIDS1 miRNA-mediated lateral inhibition controls rhizoid cell patterning in Marchantia polymorpha. *Curr. Biol.* 30, 1905-1915. 10.1016/j.cub.2020.03.032 32243863

[DEV205696C77] Tomescu, A. M. and Rothwell, G. W. (2022). Fossils and plant evolution: structural fingerprints and modularity in the evo-devo paradigm. *EvoDevo* 13, 8. 10.1186/s13227-022-00192-7 35236418 PMC8892741

[DEV205696C78] Torii, K. U. (2012). Two-dimensional spatial patterning in developmental systems. *Trends Cell Biol.* 22, 438-446. 10.1016/j.tcb.2012.06.002 22789547

[DEV205696C79] Tremblay, S. and Mercadal, J. (2026). Homology of the dark cells of Paleozoic liverworts with the specialized oil body cells of modern liverworts (Marchantiophyta). *New Phytol.* 10.1111/nph.71332 PMC13443199 42260898

[DEV205696C80] Tseng, C.-C., Woolley, T. E., Jiang, T.-X., Wu, P., Maini, P. K., Widelitz, R. B. and Chuong, C.-M. (2024). Gap junctions in Turing-type periodic feather pattern formation. *PLoS Biol.* 22, e3002636. 10.1371/journal.pbio.3002636 38743770 PMC11161087

[DEV205696C81] Van Der Valk, T., Pečnerová, P., Díez-del Molino, D., Bergström, A., Oppenheimer, J., Hartmann, S., Xenikoudakis, G., Thomas, J. A., Dehasque, M., Sağl can, E. et al. (2021). Million-year-old DNA sheds light on the genomic history of mammoths. *Nature* 591, 265-269. 10.1038/s41586-021-03224-9 33597750 PMC7116897

[DEV205696C82] Walton, J. (1925). Carboniferous bryophyta: I. hepaticae. *Ann. Bot.* 39, 563-572. 10.1093/oxfordjournals.aob.a089964

[DEV205696C83] Zimm, R., Berio, F., Debiais-Thibaud, M. and Goudemand, N. (2023). A shark-inspired general model of tooth morphogenesis unveils developmental asymmetries in phenotype transitions. *Proc. Natl Acad. Sci. USA* 120, e2216959120. 10.1073/pnas.2216959120 37027430 PMC10104537

