# Supplementary material for: Lateral inhibition governs ancestral cellular patterning in fossil and extant liverworts
Source: Development. 2026 Jul 23;153(14):dev205696. doi: 10.1242/dev.205696 (PMC13450899; doi:10.1242/dev.205696)
Supplement: Supplementary file 1 [file develop-153-205696-s1.pdf]

Table S1. Parameters used for the model with local activation and lateral inhibition, their description, and their values (in arbitrary units). The parameters used for the analysis (α_0 and γ) are shaded in gray. $\mathscr{U}[0,1]_{N_t}$ denotes a uniform distribution of random numbers between 0 and 1 in a tissue of N_t cells.

Parameter	Description	Value (a.u.)
α_0	basal transcription rate of u	variable
α	maximum value of u self-activation	2
k_u	threshold of u self-activation	1.2
n_u	cooperativity exponent of u self-activation	4
k_c	repression rate of u by v	1
d_u	u degradation	1
γ_0	basal transcription rate of v	0.1
γ	maximum value of v activation by u	variable
k_v	threshold of v activation by u	2
n_v	cooperativity exponent of v activation by u	4
d_v	v degradation	1
D_v	diffusion coefficient of v	4
u_T	threshold defining dark cell fate	1.0
u_0	initial concentration of u	$1.35 \times \mathscr{U}[0,1]_{N_t}$
v_0	initial concentration of v	$0.4 \times \mathscr{U}[0,1]_{N_t}$

Development • Supplementary information

Table S2. Table showing the minimum (x_l^{min}), maximum (x_l^{max}), and tolerance values (δ_-, δ_+) for each metric and pattern type in *M. sharonae*, and extant taxa.

**Metric**	**Patterns**	x_l^{min}	x_l^{max}	δ_-	δ_+
dark/OB cell frequency	*M. sharonae* (type I)	0.194	0.263	0	0.02
	M. sharonae (type II)	0.076	0.189	0	0
	M. sharonae (type III)	0.148	0.299	0	0
	M. sharonae (type IV)	0.005	0.024	0	0
	T. lacunosa	0.092	0.228	0	0
	A. nana	0.097	0.219	0	0
	M. polymorpha	0.033	0.045	0	0
idioblast proportion	*M. sharonae* (type I)	0.82	0.96	0.05	0.05
	M. sharonae (type II)	0.70	1	0	0
	M. sharonae (type III)	0.67	0.86	0	0
	M. sharonae (type IV)	0.91	1	0	0
	T. lacunosa	0.56	1	0	0
	A. nana	0.65	0.89	0	0
	M. polymorpha	0.94	1	0.02	0
Moran's *I*	*M. sharonae* (type I)	-0.205	-0.139	0.05	0.05
	M. sharonae (type II)	-0.176	-0.011	0	0
	M. sharonae (type III)	-0.140	-0.035	0	0
	M. sharonae (type IV)	-0.002	0.015	0.005	0.005
	T. lacunosa	-0.185	-0.015	0	0
	A. nana	-0.136	-0.052	0	0
	M. polymorpha	-0.035	-0.012	0.01	0.01
average dark/OB cell distance $\langle\lambda\rangle$	*M. sharonae* (type I)	2.13	2.53	0.3	0.3
	M. sharonae (type II)	2.45	3.87	0	0
	M. sharonae (type III)	1.96	2.52	0	0
	M. sharonae (type IV)	5.88	18.00	0	0
	T. lacunosa	2.04	3.32	0	0
	A. nana	2.09	3.28	0	0
	M. polymorpha	4.57	5.69	0.1	0.1
CV_λ	*M. sharonae* (type I)	0.25	0.35	0.05	0.05
	M. sharonae (type II)	0.26	0.50	0	0
	M. sharonae (type III)	0.34	0.47	0	0
	M. sharonae (type IV)	0	0.45	0	0
	T. lacunosa	0.27	0.48	0	0
	A. nana	0.31	0.44	0	0
	M. polymorpha	0.28	0.0.44	0.02	0.02

Development • Supplementary information

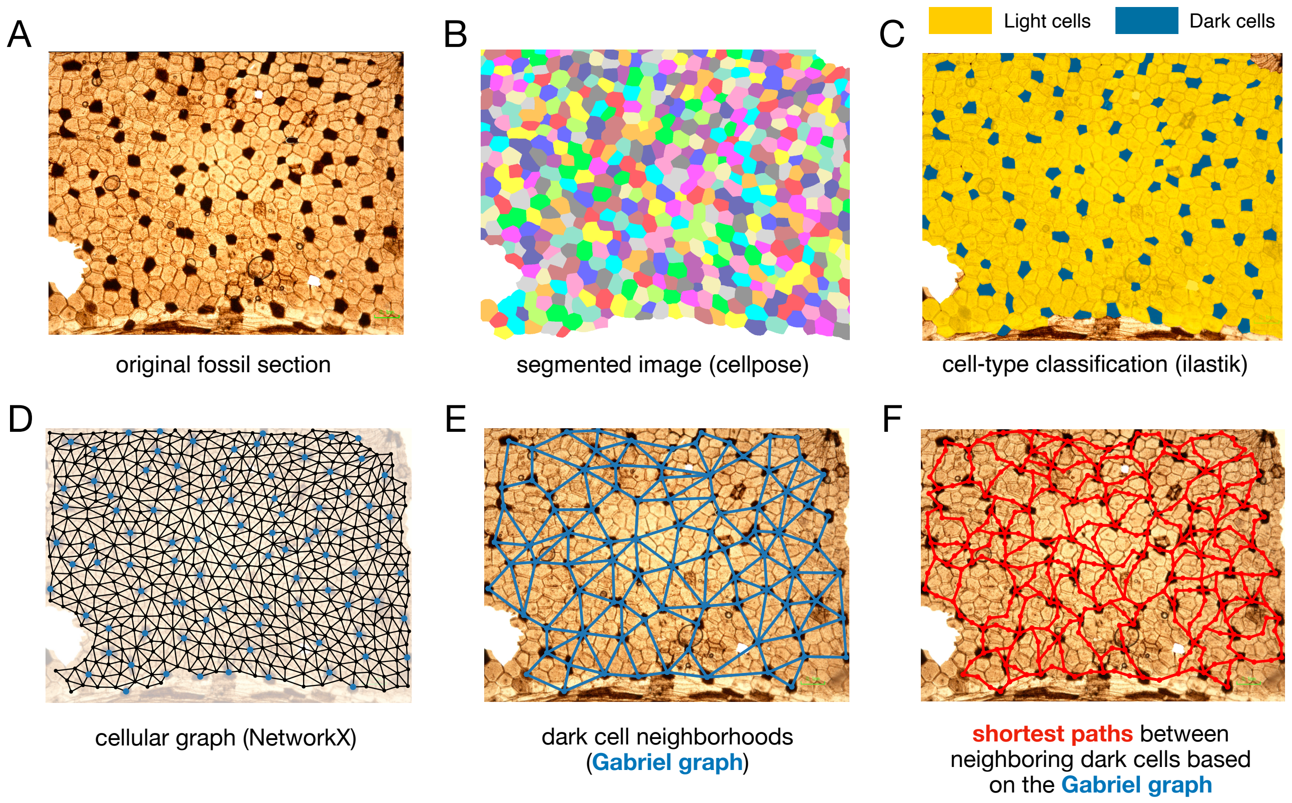

Fig. S1. Pipeline for quantifying dark/OB cell patterns. (A) Example fossil section used for segmentation, annotation, and analysis (accession number UC250153.05). (B) Cellular segmentation with `cellpose`[1], showing all segmented cells in different colors. (C) Classification of cell types (dark and light) using `ilastik`[2]. (D–F) Pipeline for spatial analysis of segmented tissues. First, we used NetworkX[3] to construct a cellular graph with edges connecting the centroids of adjacent cells (D). Then, we constructed a Gabriel graph that defines dark cell neighborhoods (E). Dark cell distances can be computed through the shortest paths between pairs of dark cells (F). The same pipeline was used for analyzing dark/OB cell patterns in the simulations.

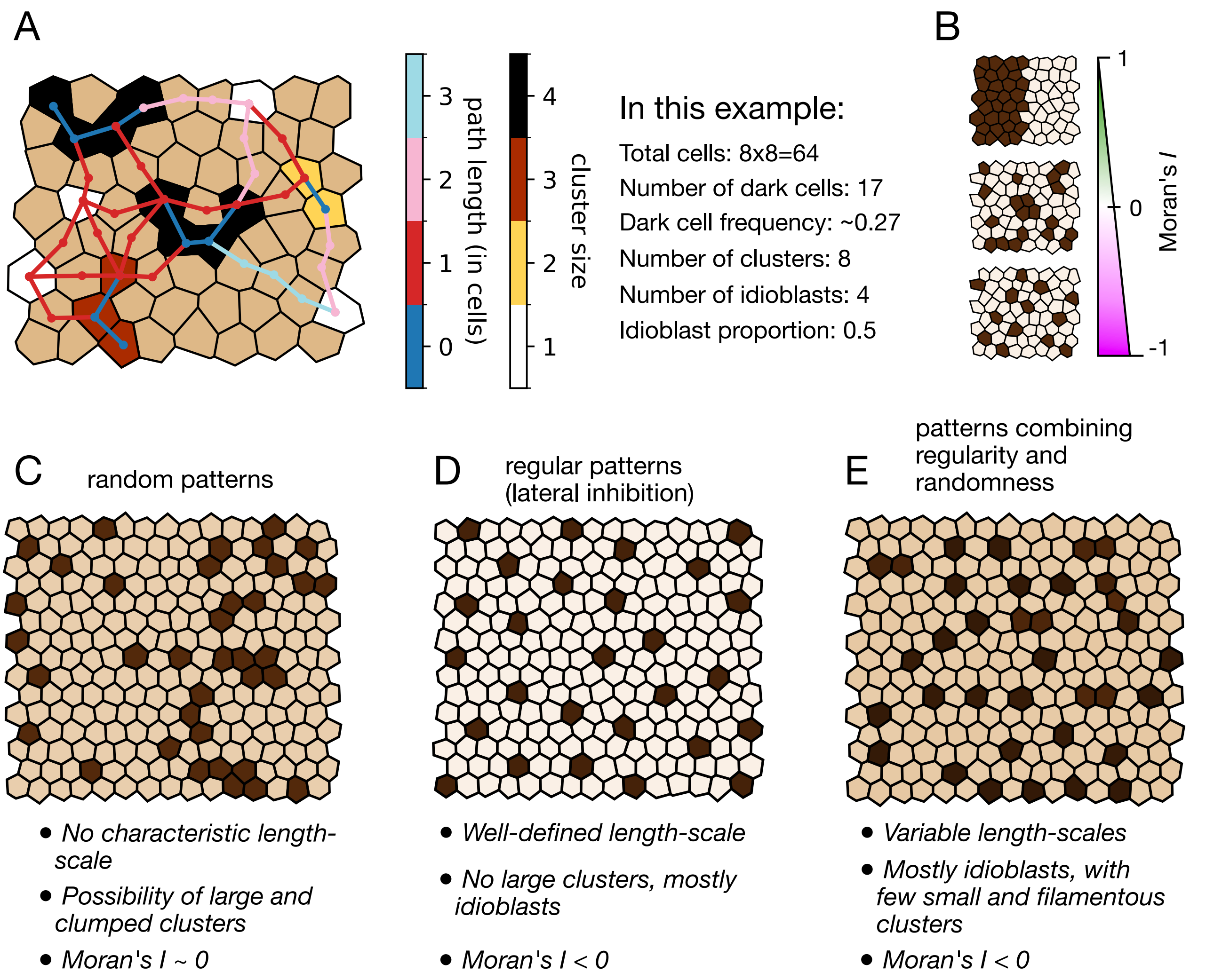

Fig. S2. Statistical metrics and their meaning for different pattern types. (A) Example tissue with several dark/OB cell clusters, showcasing cluster sizes in different colors and the shortest paths between pairs of neighboring dark cells. Note that ”dark cells” in the legend refer to OB cells, and the color of these cells denotes the cluster size—”dark cells” therefore include cells colored in black, dark brown, yellow, and white. (B) Intuitive sketch showing how Moran's I changes depending on the pattern. Completely clustered patterns have Moran's $I \approx 1$; random patterns have Moran's $I \approx 0$; regular patterns have Moran's $I \approx -1$. (C–E) Each pattern type has a statistical signature that can be used to infer the mechanisms underlying its patterning. Random patterns have no characteristic length scale, and Moran's $I \approx 0$ (C); regular patterns are associated with lateral inhibition, with a well-defined length scale, no large clusters, and negative Moran's I (D); mixed patterns are associated with a combination of randomness and lateral inhibition, with variable length scales, filamentous clusters, and negative Moran's I (E).

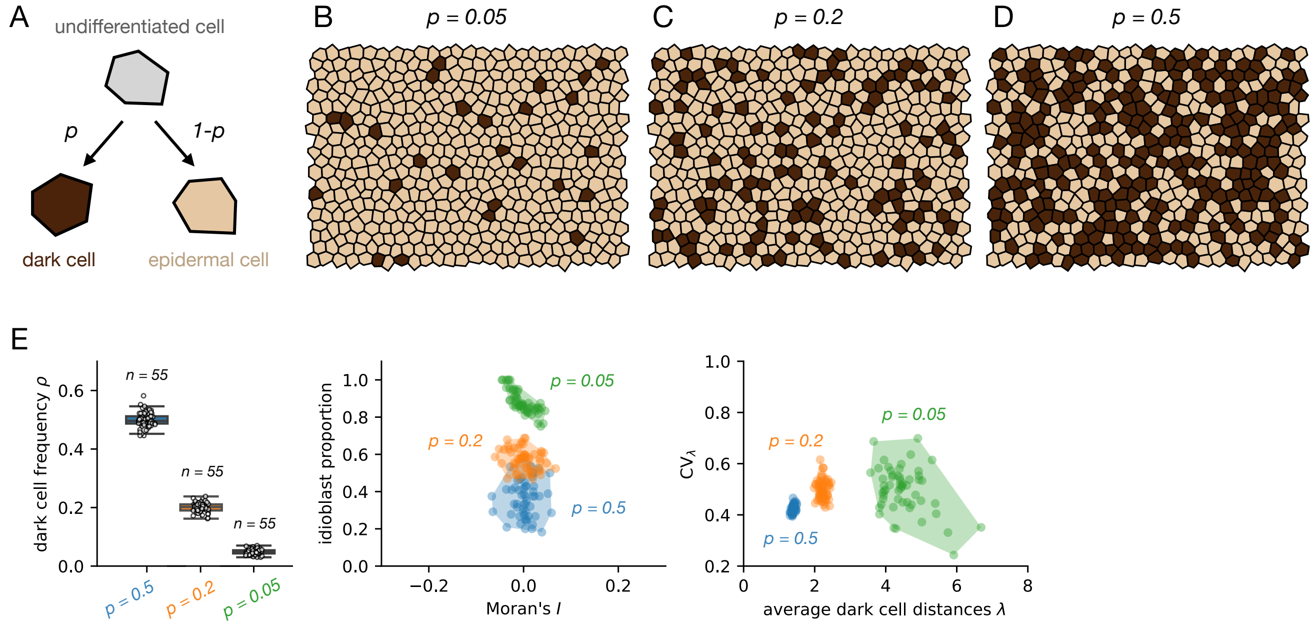

Fig. S3. Random models and their statistics. (A) In a random model of cell specification, every undifferentiated cell (light gray) can differentiate into a dark cell or an epidermal cell with probability p and $1-p$, respectively. (B–D) Typical dark cell patterns obtained with the random model, for $p = 0.05$ (B), $p = 0.2$ (C), and $p = 0.5$ (D). (E) Spatial statistics (dark cell frequency, Moran's I, idioblast proportion, average dark cell distance $\langle\lambda\rangle$, and CV_λ) of $n = 55$ patterns for each value of p detailed above. Regardless of the value p, random patterns have a Moran's I around 0, and their idioblast proportion is inversely proportional to p. The lower the p, the larger the distance between pairs of dark cells, whereas CV_λ remains approximately constant.

Development • Supplementary information

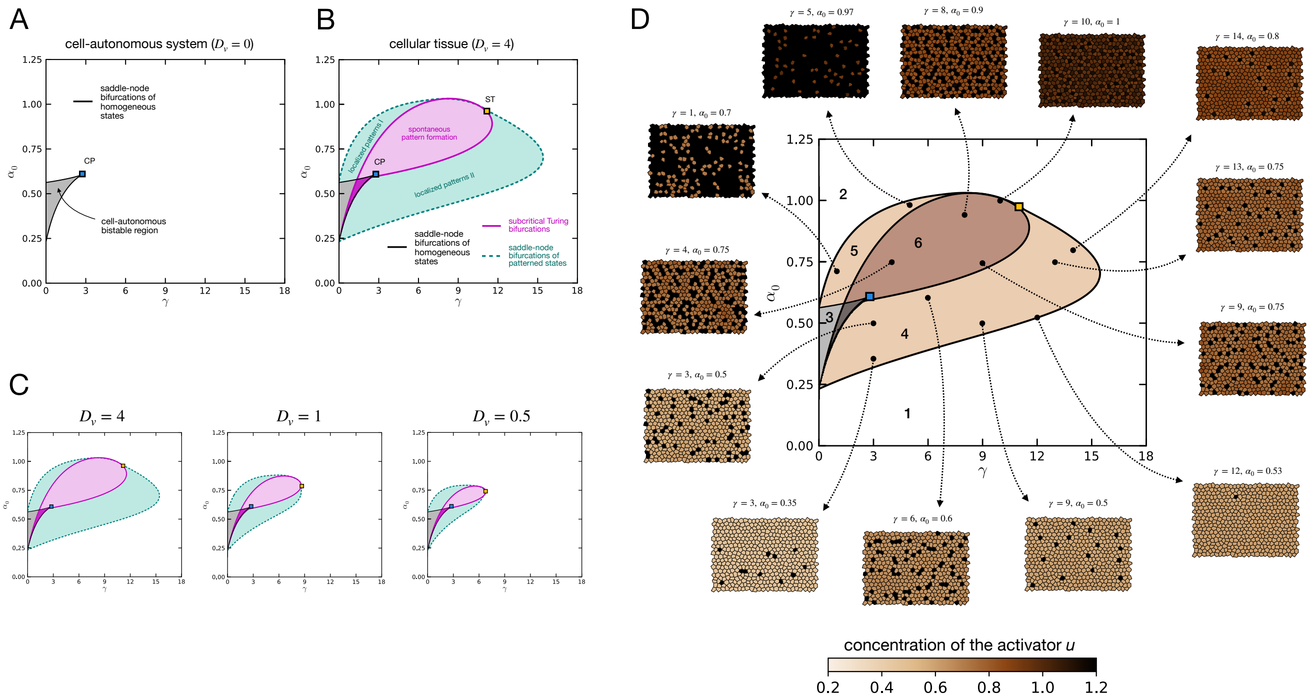

Fig. S4. Bifurcation diagrams and patterning regimes of the mathematical model. (A) Stability diagram of the mathematical model for a single cell, showing a region of cell-autonomous bistability enclosed by saddle-node bifurcations that collide in a cusp point (CP). (B) Stability diagram of the mathematical model for a cellular tissue, showing each patterning region and the bifurcations separating them. Subcritical Turing bifurcations enclose the region of spontaneous pattern formation (pink curve). Overlapping with the region of cell-autonomous bistability, there is a small region (dark pink region) where the high-u state can lose stability due to inhomogeneous perturbations. In this region, patterned states coexist with the low-u homogeneous state. The two regions of localized patterns are delimited by saddle-node bifurcations (dashed turquoise curves) associated with the states with a single light cell (localized patterns I) and the state with a 1 single dark cell (localized patterns II). The blue square denotes a cusp point (CP) where the two saddle-node bifurcations associated with the homogeneous states collide. The yellow square denotes the saddle-Turing (ST) point, where the two saddle-node bifurcations associated with the 1-cell states collide with the subcritical Turing bifurcation. (C) The effect of different diffusion coefficients ($D_v = 4, 1, 0.5$) on patterning regions. As diffusion becomes smaller, the patterning regions decrease in size. (D) Stationary dark cell patterns obtained in different parameter regions. In the region of spontaneous pattern formation, patterns exhibit spatial regularity, but their density can vary. In regions of localized patterns, multiple patterning states can coexist with the homogeneous state, enabling the formation of patterns that are restricted to specific areas rather than spanning the whole tissue. The remaining parameters and initial conditions for obtaining these patterns are detailed in Table S1.

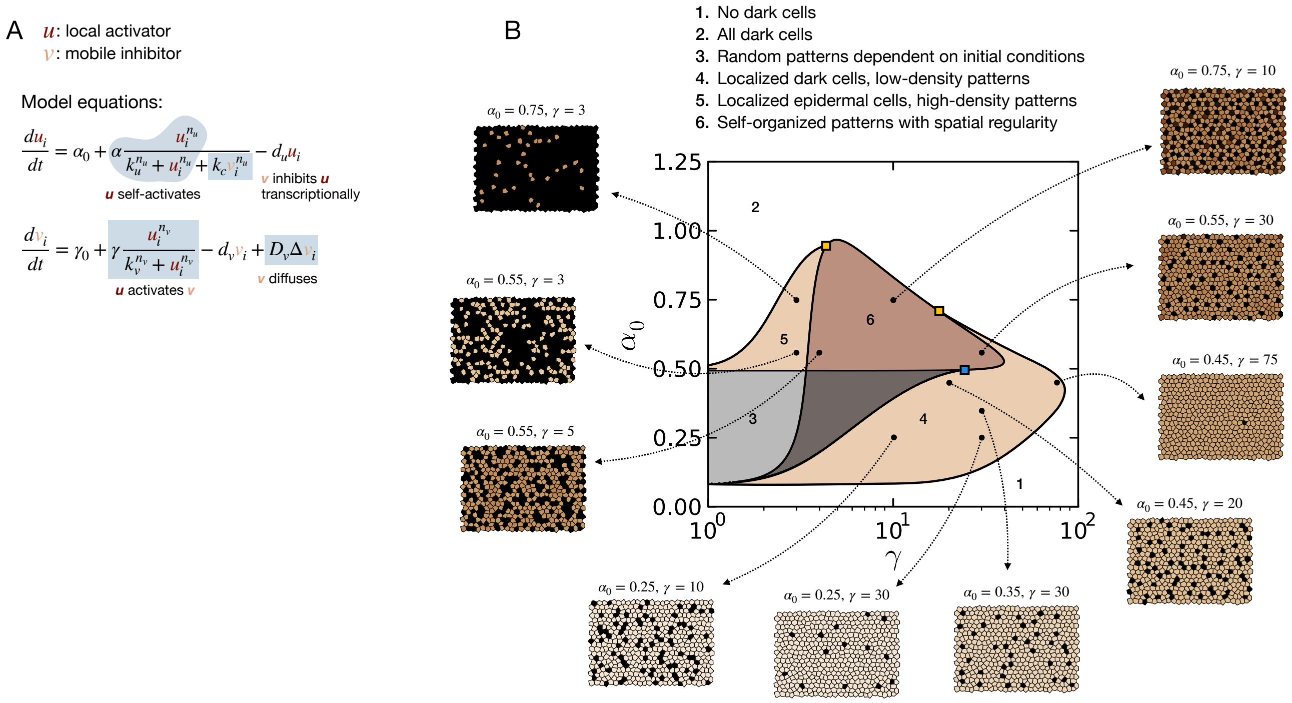

Fig. S5. Patterning regimes of an activator-inhibitor model with transcriptional inhibition. (A) Equations of the model, where u self-activates and v transcriptionally inhibits the activity of u. The remaining interactions are the same as in the original model. (B) Stability diagram showing the stationary dark cell patterns obtained in different parameter regions. This model exhibits equivalent patterning regions as the original model (bistability of homogeneous states, spontaneous pattern formation, and localized states), demonstrating that the diversity of patterns is robust to the precise implementation of inhibition. As in Fig. 3B, the blue square denotes a cusp point where two saddle-node bifurcations collide and annihilate; the yellow squares denote the collision between a subcritical Turing bifurcation and two saddle-node bifurcations. The remaining parameters and initial conditions are the same as in the original model, detailed in Table S1.

Development • Supplementary information

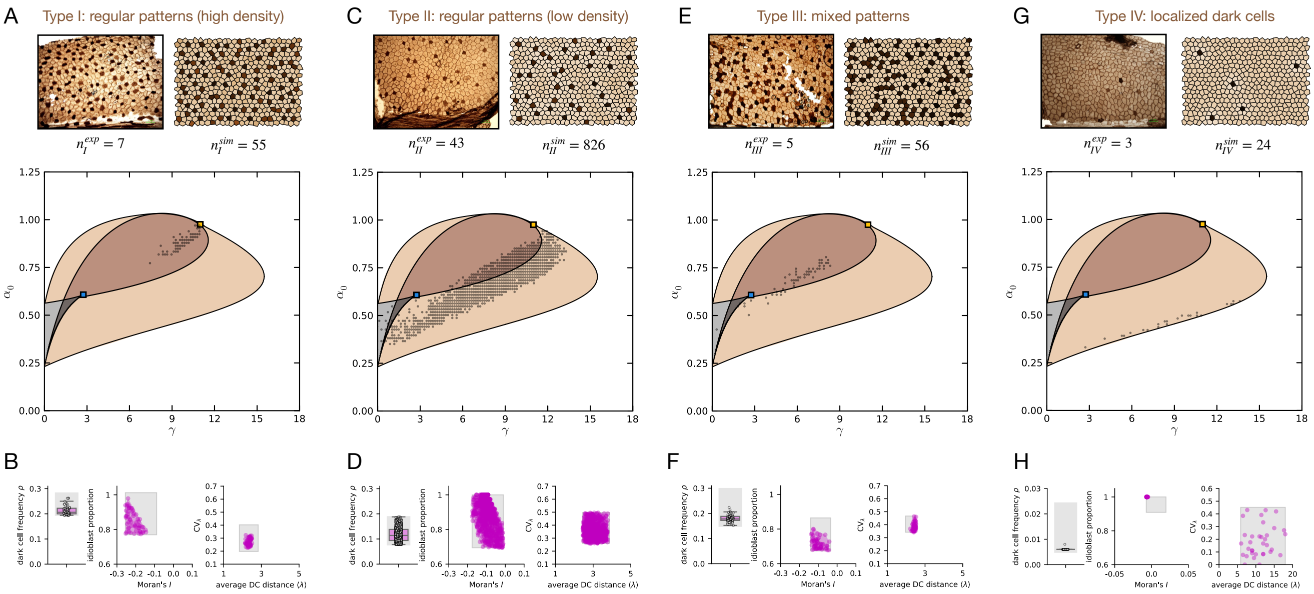

Fig. S6. Pattern types in *M. sharonae*, their associated model parameter values, and their statistical analysis. (A, B) Type I: regular, high dark cell density patterns appear in the region of spontaneous pattern formation. Due to this regularity, Moran's I is negative, and the average dark cell distance is very consistent, with small CV_λ. (C, D) Type II: Regular, low dark cell density patterns appear between the regions of spontaneous pattern formation and the region of localized patterns. These patterns are less regular than Type I, with Moran's I closer to 0 and larger CV_λ. (E, F) Type III: mixed patterns appear in the region of spontaneous pattern formation close to the bistability region. (G, H) Type IV: patterns with only a few dark cells appear only in the region of low-density localized patterns close to the bifurcation boundary. Stability diagrams are as in Fig. 3C. Black scattered points mark the values of (γ, α_0) reproducing the dark cell patterns of each type. Pink dots in panels B, D, F, and H correspond to the (γ, α_0) pairs of the associated stability diagrams. Gray boxes in panels B, D, F, and H mark the regions between $x_l^{\min} - \delta_-$ and $x_l^{\max} + \delta_+$ for each metric (see Table S2). Fossil images in panels A, C, E, and G are the same as those in Figs. 3D, E, and F.

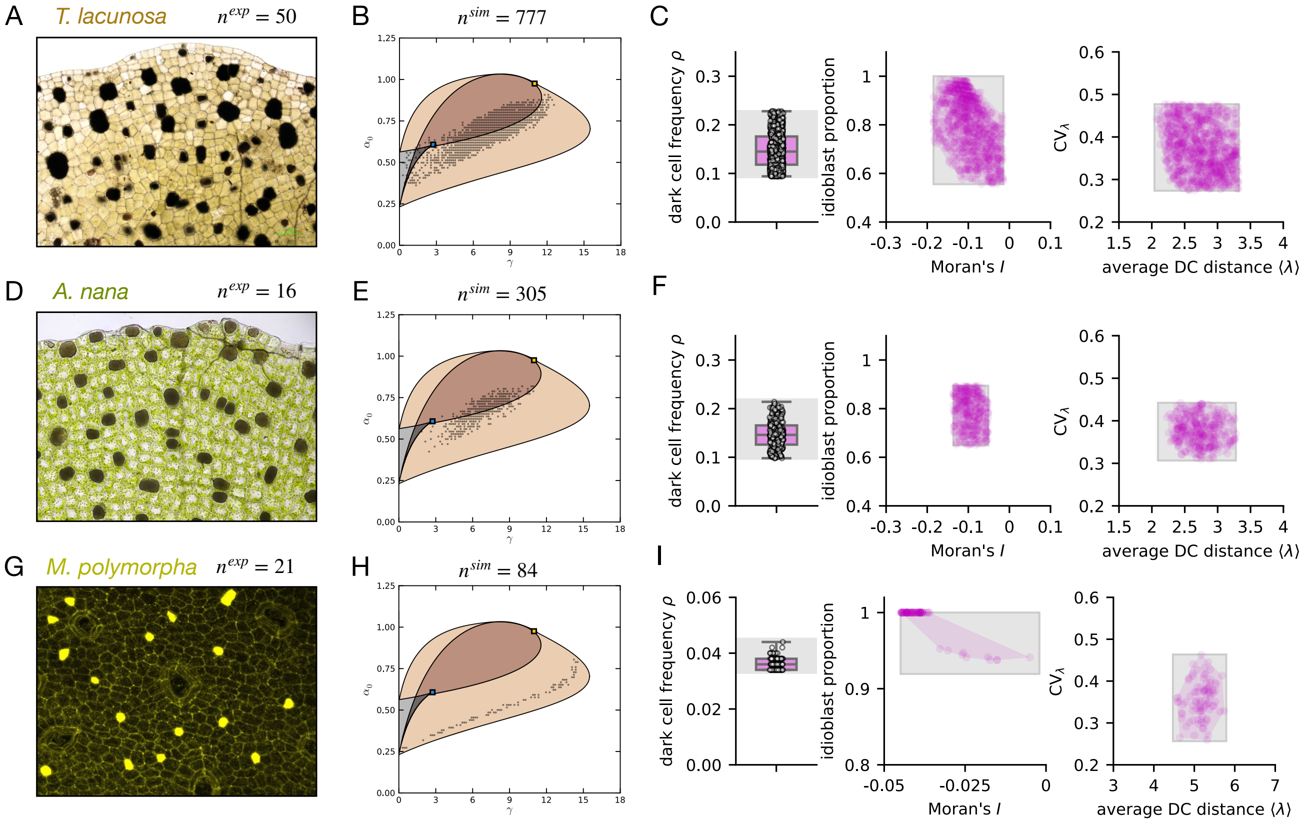

Fig. S7. Values of (γ,α_0) associated with OB cell patterns in extant liverworts. (A–C) In *T. lacunosa*, OB cell patterns appear in the regions of spontaneous and localized patterns. (D–F) Patterns in *A. nana* appear in similar regions than *T. lacunosa*, highlighting their phylogenetic similarity. (G–I) In *M. polymorpha*, OB cells appear with a very low frequency in the region of localized patterns close to the bifurcation boundary. Black scattered points mark the values of (γ,α_0) reproducing OB cell patterns in each species. Pink dots in panels C, F, and I correspond to the (γ,α_0) pairs of the associated stability diagrams. Gray boxes in C, F, and I mark the regions between $x_l^{min} - \delta_-$ and $x_l^{max} + \delta_+$ for each metric (see Table S2). The *M. polymorpha* image shown in panel G corresponds to a cropped section of Fig. 4E and rotated 90º.

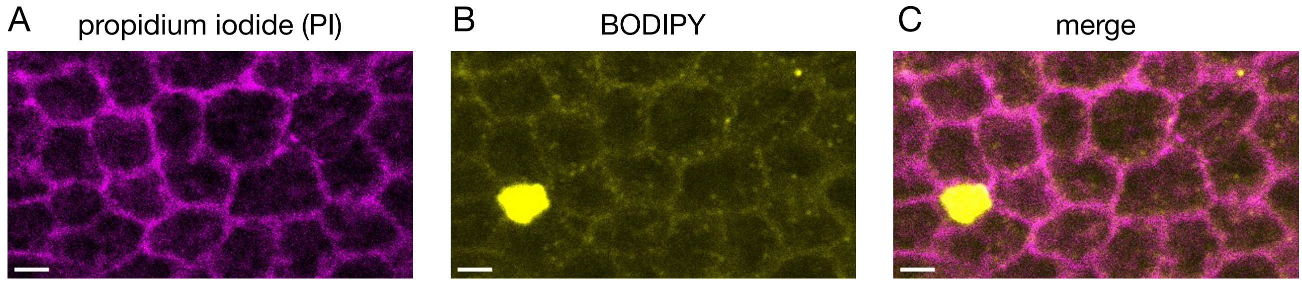

Fig. S8. BODIPY and PI staining in the lobe region of a *Marchantia* thallus. (A) Confocal image showing cell walls in the PI channel. (B) Cell boundaries seen through BODIPY staining. (C) Merge, confirming that BODIPY enables the detection of cell boundaries in the observed region. Scale bars = 10 μm.

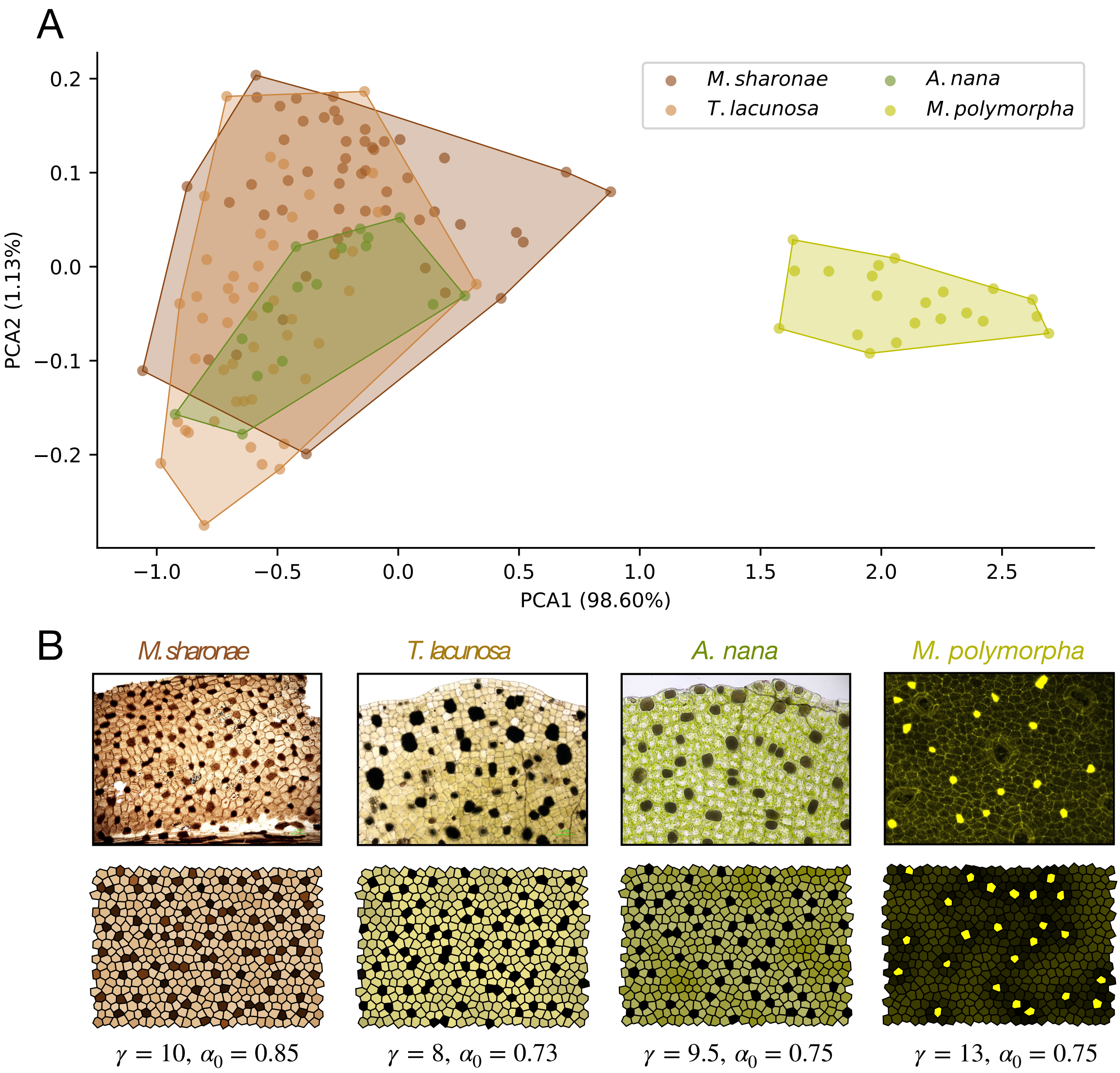

Fig. S9. OB cell distribution in different species. (A) Principal component analysis (PCA) showing how different species cluster in the space of (PCA1, PCA2). PCA was performed on the five quantification measures of the dark cells/OB patterns using the scikit-learn package in python[4]. Patterns in *M. sharonae*, *T. lacunosa*, and *A. nana* overlap in this space, showcasing their similarity. OB cell patterns in *M. polymorpha* appear far from the rest due to their significantly lower OB cell frequency. (B) Patterns (top) and associated example simulations (bottom) reproducing the spatial statistics of each species, together with the parameters (γ,α_0) used. The remaining parameters and initial conditions for the simulations are detailed in Table S1. The *M. sharonae* image in panel B is the same as Fig. 1B bottom, and images of *T. lacunosa*, *A. nana*, and *M. polymorpha* are the same as those in Fig. S7A, D, and G. The *M. polymorpha* image corresponds to a cropped section of Fig. 4E and rotated 90º.

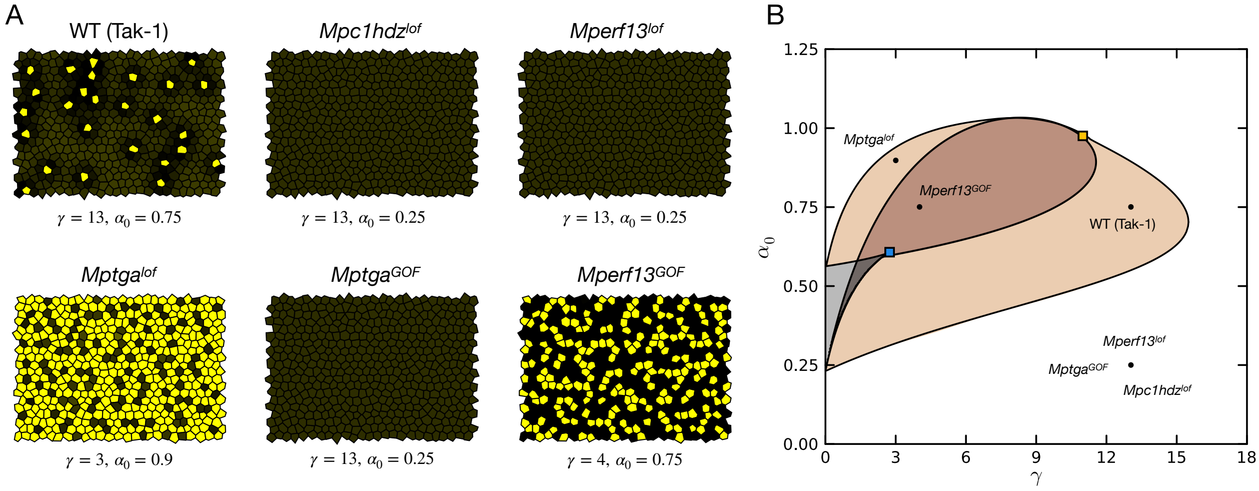

Fig. S10. The model can reproduce OB cell patterns in gain and loss-of-function genes controlling OB cell specification. (A) Different patterns obtained with the model showing the qualitative OB cell distribution in WT and different mutants. In $Mpc1hdz^{lof}$ and $Mperf13^{lof}$, OBs do not appear due to the lack of their activators. In $Mptga^{lof}$, the number of OBs increases dramatically, as TGA is an inhibitor of OB cell specification. This increase also occurs in $Mperf13^{GOF}$, but more weakly. (B) Parameter values used for the simulations in A. In our framework, different mutants reside in different regions of the parameter space, therefore exhibiting different OB cell patterns. Parameters in the mutants were chosen to qualitatively match the observed patterns. In $Mpc1hdz^{lof}$ and $Mperf13^{lof}$, the loss of activator activity was implemented by reducing α_0 with respect to the WT, thereby shifting the system out of the patterning regime. The same approach was used for $Mptga^{GOF}$, where overexpression is assumed to inhibit the activator activity. For $Mperf13^{GOF}$ and $Mptga^{lof}$, parameters were adjusted to increase OB cell density while ensuring that not all cells adopt OB identity. The remaining parameters and initial conditions are detailed in Table S1.

Development • Supplementary information

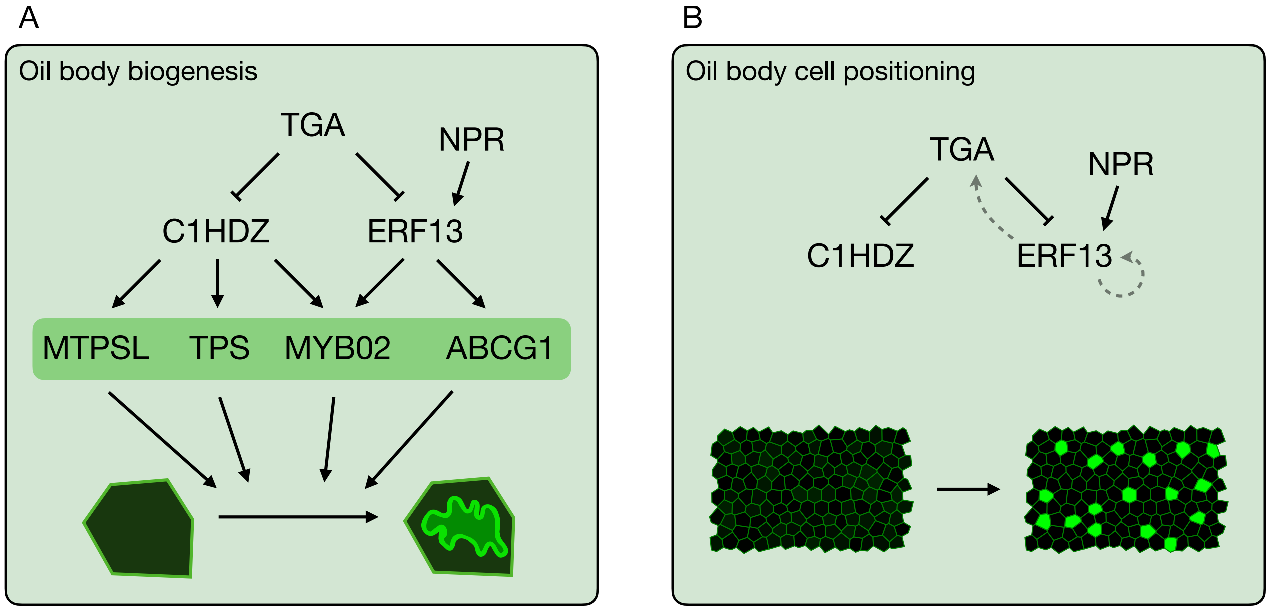

Fig. S11. Gene regulatory network controlling OB biogenesis and positioning. (A) Known regulatory pathway controlling OB biogenesis. (B) Transcription factors controlling the spatial distribution of OB cells and hypothetical feedback interactions required to generate cell-type patterns. Normal arrows represent activation, and blunt arrows represent inhibition.

Supplementary References

[1] C. Stringer, T. Wang, M. Michaelos, and M. Pachitariu, "Cellpose: a generalist algorithm for cellular segmentation," *Nature methods*, vol. 18, no. 1, pp. 100–106, 2021.

[2] S. Berg, D. Kutra, T. Kroeger, C. N. Straehle, B. X. Kausler, C. Haubold, M. Schiegg, J. Ales, T. Beier, M. Rudy, *et al.*, "Ilastik: Interactive Machine Learning for (bio) Image Analysis," *Nature Methods*, vol. 16, no. 12, pp. 1226–1232, 2019.

[3] A. Hagberg, P. J. Swart, and D. A. Schult, "Exploring Network Structure, Dynamics, and Function Using NetworkX," tech. rep., Los Alamos National Laboratory (LANL), Los Alamos, NM (United States), 2008.

[4] F. Pedregosa, G. Varoquaux, A. Gramfort, V. Michel, B. Thirion, O. Grisel, M. Blondel, P. Prettenhofer, R. Weiss, V. Dubourg, *et al.*, "Scikit-learn: Machine learning in python," *the Journal of machine Learning research*, vol. 12, pp. 2825–2830, 2011.
