# Supplementary material for: Lateral inhibition governs ancestral cellular patterning in fossil and extant liverworts
Source: Development. 2026 Jul 23;153(14):dev205696. doi: 10.1242/dev.205696 (PMC13450899; doi:10.1242/dev.205696)
Supplement: Supplementary file 2 [file develop-153-205696_review_history.pdf]

Lateral inhibition governs ancestral cellular patterning in fossil and extant liverworts

Josep Mercadal, Susan Tremblay, Leonie Kraska, Martin A. Hutten and Pau Formosa-Jordan
DOI: 10.1242/dev.205696

Editor: Dominique Bergmann

Review timeline

Original submission:	26 March 2026
Editorial decision:	8 May 2026
First revision received:	10 June 2026
Accepted:	11 June 2026

Original submission

First decision letter

MS ID#: dev.205696

MS TITLE: Lateral inhibition governs ancestral cellular patterning in fossil and extant liverworts

AUTHORS: Josep Mercadal, Susan Tremblay, Leonie Kraska, Martin A. Hutten and Pau Formosa-Jordan

Dear Dr Mercadal,

I have now received all the referees reports on the above manuscript, and have reached a decision. The referees' comments are appended below.

The overall evaluation is positive and we would like to publish a revised manuscript in Development, provided that the referees' comments can be satisfactorily addressed. Please attend to all of the reviewers' comments in your revised manuscript and detail them in your point-by-point response. If you do not agree with any of their criticisms or suggestions explain clearly why this is so. If it would be helpful, you are welcome to contact us to discuss your revision in greater detail. Please send us a point-by-point response indicating your plans for addressing the referees' comments, and we will look over this and provide further guidance.

Reviewer 1

SUMMARY OF THE ADVANCE MADE IN THIS PAPER AND ITS POTENTIAL SIGNIFICANCE TO THE FIELD

The paper combines studies of computational modelling with anatomical data from fossil and living liverworts to propose that idioblasts are patterned by a lateral inhibition mechanism.
The work makes predictions that can be tested experimentally in the future and showcases a novel interdisciplinary approach to study the evolution of development in plants.

Overview
I really enjoyed reading the paper. It was well written and very well illustrated. The figures did a great job of making the fossil data and modelling approach very accessible to a general audience. I thought the approach was novel and I hope it catalyses more studies trying to integrate fossil data into theories on the evolution of development.

© 2026. Published by The Company of Biologists under the terms of the Creative Commons Attribution License (https://creativecommons.org/licenses/by/4.0/).

The fossil data included in the study is exceptional. The preservation of the samples is excellent and the sample size is enormous for a study on a single fossil species. This large sample size is important as it captures the natural diversity present in the fossils, and adds robustness to the comparison with the modelling approach.
I found the description of the methods and modelling were very clear, first moving from the direct comparison with a random distribution of idioblasts to a more complex model of lateral inhibition. I think the finding that the fossil showed a non-random distribution is already a major finding and will hopefully be a template for other investigations of development in fossils for the future. However, I the authors then significantly extend the scope of the investigation to investigate extant species. Although data currently available from Marchantia polymorph suggests that the patterning mechanism was likely much more complex than the simulation here, I still think this is a very valuable addition and will be used to can hope frame specific genetic hypotheses to test experimentally in the future.
I thanks the author for their interesting and interdisciplinary study and approach.

SUGGESTIONS TO AUTHORS

1. I think slightly more could have been added to discuss the overall distribution and variation of idioblasts between specimens. E.g. it may be predicted that distribution varied on an individual thallus - e.g. higher or lower numbers at the midrib than the peripheral regions. Is there evidence that a single specimen / individual shows a change in density within a specimen or are they all quite easily separated into major categories. Are there any other cellular differences between specimens that exhibit a very low frequency (category, type 4).
2. When investigating M. polymorpha what impact does the development of airpores have or your results or interpretation? Superficially it looks like air pore development also inhibit or at least influence the formation of oil bodies.
3. The work generated over 600 permanent fossils slides. However, it is not clear where these are accessioned, and it would be good to add specimen accession codes in the figure captions for all figured material.
4. In your information covering the collection of Treubia do you have more information about the location or permits for collecting. Or details saying why this information isn't provided - e.g. there is no requirement to have a permit to collect in the area you investigated.

Specific comments
Line 21: " where genetic material is rarely preserved" - I think you can be more specific here and demonstrate that ancient DNA has been reported for samples less than 1 million years, but beyond that reports get very ambiguous. Leaving almost the entire fossil record inaccessible to studies of genetic material.
Line 75: "strong excess of idioblasts (clusters of size k = 1)" - for a general audience I think it might be worth stating what cluster size 1 means in lay terms - e.g. a single dark cell surrounded on all sides by light cells, and maybe referring to Figure S2 to illustrate this.
Line 92-93: "preventing adjacent dark cells from adopting the same fate" - your data shows that clusters do form so I don't think you can say the process will "prevent". It sounds like it reduces the chance this will happen.
Line 170: "to a deeply conserved developmental mechanism" maybe add that this is currently undescribed, or uncharacterised.
Line 180-181: When referring to the Late Silurian or Late Ordovician - it might be worth adding a time in millions of years.
Line 205: "could even predate the divergence of bryophyte lineages", maybe also add that the alternative is they have evolved independently in mosses.
Line 220: "Despite the lack of any feedback mechanism that could function as an activator inhibitor system capable of cell-type patterning". Is it possible to deal with this more specifically and say why this doesn't undermine your predictions of a highly conserved mechanism. E.g. highlighting other examples where lateral inhibition functions within a complex genetic network.

<u>Reviewer 2</u>

SUMMARY OF THE ADVANCE MADE IN THIS PAPER AND ITS POTENTIAL SIGNIFICANCE TO THE FIELD

© 2026. Published by The Company of Biologists under the terms of the Creative Commons Attribution License (https://creativecommons.org/licenses/by/4.0/).

In this manuscript, the authors describe the frequency and distribution of liverwort oil body cells in both fossil and extant species. They show that the various patterns of these cells can be reproduced with a lateral inhibition model, where a model of random cell fate assignment does not.
The manuscript is well-written; I have only one major point, specified below, and a number of smaller corrections.

SUGGESTIONS TO AUTHORS

Major point:
The case for lateral inhibition is compelling, given its ability to produce a variety of different patterns. I would still like to see a discussion on possible alternatives (and why those would/would not work) -- in many cases in biology, patterns can be created in several different ways, so similarity alone may not be enough.
Furthermore, the authors have chosen for the inhibitor to degrade the activator with a mass-action term, but the potential components of the patterning mechanisms listed in the discussion and figure S10 are all transcription factors. Do you expect results to meaningfully change if the inhibitor were a repressing TF?

Medium point:
I suspect your formulation of Moran's I statistic in the methods is not quite right: For a fully segregated tissue to yield a value of 1, surely the w matrix should correspond to some inverse of distance? In most definitions of the statistic that I have found, w is a weight matrix for distance, not distance itself (line 489).

Minor points:
- pg 2, lines 38, 41, 44: a bit of repetition of the "phenomenon/hallmark of lateral inhibition".
- I could not find reference to figure 2c in the text
- Something appears to have gone wrong with the color bar of figure S4D.
- Are the magenta dot plots in S5 and 6 made with a single set of parameter values? If so, could you specify that in the legend - it was a bit hidden in the methods.
- ln 164: wonky sentence, probably just remove "what".
- Could you discuss whether you think that Jungermanniopsida retain the lateral inhibition mechanism, given the lack of species with scattered cells?
- In a similar vein, you mention in the discussion (line 198-205) that "the internal and spatial variability in OB cells is taxonomically important and could offer insights into the adaptive strategies of early land plants." Given that your model suggests that changes in patterning can result from straightforward shifts in parameters, how reliable would such a taxonomy be over longer evolutionary time scales?
- fig S9: Can you explain in the legend the choices for mutant parameters?

First revision

Author response to reviewers' comments

Reviewer 1: SUMMARY OF THE ADVANCE MADE IN THIS PAPER AND ITS POTENTIAL SIGNIFICANCE TO THE FIELD

The paper combines studies of computational modelling with anatomical data from fossil and living liverworts to propose that idioblasts are patterned by a lateral inhibition mechanism. The work makes predictions that can be tested experimentally in the future and showcases a novel interdisciplinary approach to study the evolution of development in plants.

Overview

I really enjoyed reading the paper. It was well written and very well illustrated. The figures did a great job of making the fossil data and modelling approach very accessible to a general

© 2026. Published by The Company of Biologists under the terms of the Creative Commons Attribution License (https://creativecommons.org/licenses/by/4.0/).

audience. I thought the approach was novel and I hope it catalyses more studies trying to integrate fossil data into theories on the evolution of development. The fossil data included in the study is exceptional. The preservation of the samples is excellent and the sample size is enormous for a study on a single fossil species. This large sample size is important as it captures the natural diversity present in the fossils, and adds robustness to the comparison with the modelling approach.

I found the description of the methods and modelling were very clear, first moving from the direct comparison with a random distribution of idioblasts to a more complex model of lateral inhibition. I think the finding that the fossil showed a non-random distribution is already a major finding and will hopefully be a template for other investigations of development in fossils for the future. However, I the authors then significantly extend the scope of the investigation to investigate extant species. Although data currently available from Marchantia polymorph suggests that the patterning mechanism was likely much more complex than the simulation here, I still think this is a very valuable addition and will be used to can hope frame specific genetic hypotheses to test experimentally in the future. I thanks the author for their interesting and interdisciplinary study and approach.

We thank the reviewer for the praise and we are happy that they value the interdisciplinary approach.

SUGGESTIONS TO AUTHORS

1. I think slightly more could have been added to discuss the overall distribution and variation of idioblasts between specimens. E.g. it may be predicted that distribution varied on an individual thallus - e.g. higher or lower numbers at the midrib than the peripheral regions. Is there evidence that a single specimen / individual shows a change in density within a specimen or are they all quite easily separated into major categories. Are there any other cellular differences between specimens that exhibit a very low frequency (category, type 4).

We agree that the overall description of dark cells in *M. sharonae* was limited. To address this, we extended our discussion and also referenced previous studies describing the morphology of the fossils (Lines 58-66).

2. When investigating M. polymorpha what impact does the development of airpores have or your results or interpretation? Superficially it looks like air pore development also inhibit or at least influence the formation of oil bodies.

This is a very interesting question that we did not address in the original manuscript. Typically, in a mature thallus, both patterns coexist, and oil body cells appear in the spaces between air pores (see Fig. 4E). Oil body cells in the thallus start emerging around day ~3, approximately coinciding with the onset of air pore formation. Since both structures are specified near the apical notch and are subsequently displaced during thallus growth, their developmental trajectories are spatially linked from an early stage. Notably, both oil body cells and air pores seem to form non-random spatial patterns. It is likely that there is some degree of spatial competition or mutual exclusion between these two structures, such that the location of one influences the location of the other through mutual inhibitory interactions. However, we do not know anything about how this interplay occurs.

That said, while the presence of air pores seemingly affects the spatial statistics of oil body cell patterns—for example, oil body cells are on average more separated that they would be without air pores—we do not think this affects our main interpretation of lateral inhibition patterning. The key observation is that almost all oil body cells appear as isolated idioblasts, and that their spatial distribution is not consistent with random cell specification. This strongly suggests that lateral inhibition is acting independently of the presence of air pores. Even in the hypothetical absence of air pores, the overall pattern would still be consistent with lateral inhibition.

We mention now the presence of air pores and its potential influence on patterning in the revised manuscript (Lines 174-178).

© 2026. Published by The Company of Biologists under the terms of the Creative Commons Attribution License (https://creativecommons.org/licenses/by/4.0/).

3. The work generated over 600 permanent fossils slides. However, it is not clear where these are accessioned, and it would be good to add specimen accession codes in the figure captions for all figured material.

We apologize for this omission. The *M. sharonae* fossils are housed in the University of California Museum of Paleontology at the University of California, Berkeley. Accession numbers for the specimens used in the experiments reported in this paper are:

UC250157.01-UC250157.06, UC250156.01-UC250156.23, UC250154.01, UC250155.01- UC250155.29, UC250153.01-UC250153.10, UC250151.01-UC250151.18, UC250152.01- UC250152.05, UC250150.01-UC250150.18, and UC250149.01-UC250149.10

We added the accession numbers of all images shown in the figures in their respective captions. We also updated the Materials and Methods section to reference the accession numbers. We also updated the Data and materials availability section to include a reference to where all the images (both of fossils and extant species) used for the analysis can be accessed: https://doi.org/10.5281/zenodo.18254721.

Unfortunately, the full-size specimen of Figure 1A is shown floating in water and could not be preserved in a permanent slide.

4. In your information covering the collection of Treubia do you have more information about the location or permits for collecting. Or details saying why this information isn't provided - e.g. there is no requirement to have a permit to collect in the area you investigated.

Yes, permits were required for all *T. lacunosa* collections, which can be provided upon request (permit number 35273-FLO). Voucher specimens of *T. lacunosa* have been deposited in the University Herbarium at UC Berkeley (accession numbers UC2036933, UC2036941, UC2036942, UC2036945, and UC2036946). We have updated the Materials and methods section to mention more explicitly the location, permits, and accession numbers.

Specific comments

Line 21: " where genetic material is rarely preserved" - I think you can be more specific here and demonstrate that ancient DNA has been reported for samples less than 1 million years, but beyond that reports get very ambiguous. Leaving almost the entire fossil record inaccessible to studies of genetic material.

We agree with the reviewer that we could be more precise in our wording. We modified the sentence accordingly (Lines 21-23) and cited two landmark papers where the oldest DNA (mammoth specimens and environmental DNA) has been recovered up to date (Refs. 4 and 5):

- [4] Van Der Valk, T., Pečnerová, P., Díez-del-Molino, D., Bergström, A., Oppenheimer, J., Hartmann, S., ... & Dalén, L. (2021). Million-year-old DNA sheds light on the genomic history of mammoths. *Nature*, *591*(7849), 265-269.

- [5] Kjær, K. H., Winther Pedersen, M., De Sanctis, B., De Cahsan, B., Korneliussen, T. S., Michelsen, C. S., ... & Willerslev, E. (2022). A 2-million-year-old ecosystem in Greenland uncovered by environmental DNA. *Nature*, *612*(7939), 283-291.

Line 75: "strong excess of idioblasts (clusters of size k = 1)" - for a general audience I think it might be worth stating what cluster size 1 means in lay terms - e.g. a single dark cell surrounded on all sides by light cells, and maybe referring to Figure S2 to illustrate this.

A clarification has been added and Fig. S2D has been cited (Lines 85-87).

Line 92-93: "preventing adjacent dark cells from adopting the same fate" - your data shows that clusters do form so I don't think you can say the process will "prevent". It sounds like it reduces the chance this will happen.

© 2026. Published by The Company of Biologists under the terms of the Creative Commons Attribution License (https://creativecommons.org/licenses/by/4.0/).

We agree that "preventing" is too strong a word. The full sentence has been changed to:

"This suggests that the mechanism of dark cell specification in *M. sharonae* was not random—as would be expected by a cell-autonomous process—but likely involved cell-to-cell interactions via lateral inhibition, **reducing the occurrence of adjacent dark cells** and generating the observed spatial regularity." (Lines 101-104)

Line 170: "to a deeply conserved developmental mechanism" maybe add that this is currently undescribed, or uncharacterised.

We added "to **an as-yet-unidentified**, deeply conserved developmental mechanism" (Line 188).

Line 180-181: When referring to the Late Silurian or Late Ordovician - it might be worth adding a time in millions of years.

Thanks for pointing this out. We have added the corresponding dates (Lines 201-202). We have also expanded this section to discuss the ancestral state of OBs in the most recent common liverwort ancestor (Lines 197-203). Because of this, we have added the following reference (Ref. 58):

- [58] Flores, J. R., Bippus, A. C., Suárez, G. M., & Hyvönen, J. (2021). Defying death: incorporating fossils into the phylogeny of the complex thalloid liverworts (Marchantiidae, Marchantiophyta) confirms high order clades but reveals discrepancies in family-level relationships. *Cladistics*, *37*(3), 231-247.

Line 205: "could even predate the divergence of bryophyte lineages", maybe also add that the alternative is they have evolved independently in mosses.

We modified and expanded this section to clarify competing hypotheses (Lines 236-243).

Line 220: "Despite the lack of any feedback mechanism that could function as an activator inhibitor system capable of cell-type patterning". Is it possible to deal with this more specifically and say why this doesn't undermine your predictions of a highly conserved mechanism. E.g. highlighting other examples where lateral inhibition functions within a complex genetic network.

Thanks for pointing this out. We added a section in the discussion (also addressing a comment of Reviewer 2) discussing other examples of lateral inhibition controlling cell fates in plants (Lines 257-270), and why similar mechanisms might operate for oil body cells despite the current lack of a known molecular mechanism.

Because of this modification, we added two new references to the manuscript (Refs. 74 and 75):

- [74] Schellmann, S., Schnittger, A., Kirik, V., Wada, T., Okada, K., Beermann, A., ... & Hülskamp, M. (2002). TRIPTYCHON and CAPRICE mediate lateral inhibition during trichome and root hair patterning in Arabidopsis. *The EMBO journal*, *21*(19), 5036-5046.

- [75] Lee, M. M., & Schiefelbein, J. (2002). Cell pattern in the Arabidopsis root epidermis determined by lateral inhibition with feedback. *The Plant Cell*, *14*(3), 611-618.

Reviewer 2: SUMMARY OF THE ADVANCE MADE IN THIS PAPER AND ITS POTENTIAL SIGNIFICANCE TO THE FIELD

In this manuscript, the authors describe the frequency and distribution of liverwort oil body cells in both fossil and extant species. They show that the various patterns of these cells can be reproduced with a lateral inhibition model, where a model of random cell fate assignment does not. The manuscript is well-written; I have only one major point, specified below, and a number of smaller corrections.

© 2026. Published by The Company of Biologists under the terms of the Creative Commons Attribution License (https://creativecommons.org/licenses/by/4.0/).

We thank the reviewer for their comments on the manuscript. Below, we address the major point and the minor corrections in detail.

SUGGESTIONS TO AUTHORS

Major point:

The case for lateral inhibition is compelling, given its ability to produce a variety of different patterns. I would still like to see a discussion on possible alternatives (and why those would/would not work) -- in many cases in biology, patterns can be created in several different ways, so similarity alone may not be enough.

We agree that there are many ways to generate periodic patterns. However, regardless of the specific mechanism, pattern formation processes in biology typically rely on some form of lateral inhibition, whether through diffusive signals (as in our proposal), through cell-to-cell contact (via ligand-receptor intercellular complexes as in Notch signaling in animal cells) or mechanical (through local changes in tension and tissue contractility). Although the molecular mechanisms can be diverse, the observed avoidance of adjacent dark/oil body cells is consistent with the general principle of lateral inhibition, regardless of the specific molecular mechanism. When we refer to "a lateral inhibition mechanism", we are not only referring to the inhibition between adjacent cells, but also the ability of this inhibition to induce a pattern-forming instability, leading to regular, periodic patterns. Given the limited knowledge we have on the factors controlling oil body cell differentiation, we believe the proposed reaction-diffusion mechanism constitutes a parsimonious phenomenology based on the limited molecular knowledge of oil body differentiation. In plants, similar reaction-diffusion mechanisms have been proposed to explain the specification of other epidermal cell types, such as rhizoids in *M. polymorpha* (Thamm, A., *et al.* (2020). *Current Biology*, *30*(10), 1905-1915; Mercadal, J., *et al.* (2026). *Cell Reports*, *45*(1)), and trichomes (Bouyer, D., *et al.* (2008). *PLoS biology*, *6*(6), e141; Balkunde, R., *et al.* (2020). *Cell Reports*, *33*(11).).

Because this question was related to a similar question by Reviewer 1, we added a section in the Discussion to clarify these points (Lines 257-270).

Furthermore, the authors have chosen for the inhibitor to degrade the activator with a mass-action term, but the potential components of the patterning mechanisms listed in the discussion and figure S10 are all transcription factors. Do you expect results to meaningfully change if the inhibitor were a repressing TF?

We agree that given the knowledge we have about the factors controlling oil body cell specification in *M. polymorpha*, it is also possible that the inhibition occurs at a transcriptional level. To this end, we tested whether transcriptional inhibition would also give similar results. To minimize changes of the original model and keep the same number of parameters, we considered the following equations:

$$\frac{du}{dt} = \alpha_0 + \alpha \frac{u^{n_u}}{k_u^{n_u} + u^{n_u} + k_c\, v^{n_u}} - d_u\, u$$
$$\frac{dv}{dt} = \gamma_0 + \gamma \frac{u^{n_v}}{k_v^{n_v} + u^{n_v}} - d_v\, v$$

which includes the transcriptional inhibition term $k_c\, v^{n_u}$ in the denominator of the regulatory function modulating the production of u, instead of the original mass-action term $-k_c\, uv$. This new term can be thought of as a transcriptional repression of u by v, occurring when v can bind to the same promoter as u. Therefore, the parameter k_C modulates the strength of the transcriptional repression. We generated a stability diagram for this model, shown in the new Supplementary Figure Fig. S5. Our results show that the regions obtained with this new model are equivalent to those of the original model, and therefore, the same types of patterns can be

© 2026. Published by The Company of Biologists under the terms of the Creative Commons Attribution License (https://creativecommons.org/licenses/by/4.0/).

obtained (Figs. 3, S4, and S5).

This similarity reflects the fact that the qualitative behavior of the system is robust to the precise mechanistic implementation of inhibition, as long as the regulatory logic is preserved (i.e., increasing *v* reduces the production of *u*). This does not mean that we are completely free in choosing the inhibitory function (or any regulatory function for that matter), as any choice is associated to specific nonlinear constraints and saturation behaviors, which can affect patterning outcomes. In particular, transcriptional regulation through Hill functions and mass-action differ in their limiting behavior at high concentrations and their sensitivity to parameter changes, which may be relevant outside the parameter regime explored here. We added a part in the manuscript to clarify this point (Lines 135-138).

Due to the addition of Fig. S5, we renumbered Figs. S6-S11.

Medium point:

I suspect your formulation of Moran's I statistic in the methods is not quite right: For a fully segregated tissue to yield a value of 1, surely the w matrix should correspond to some inverse of distance? In most definitions of the statistic that I have found, w is a weight matrix for distance, not distance itself (line 489).

We thank the reviewer for pointing this out. In our implementation, the weights *w_{ij}* do not represent distances between cells, but rather a spatial weight matrix defining cell adjacency, such that *w_{ij}=1* when cells *i* and *j* are neighbors and *w_{ij}=0* otherwise. We described it incorrectly in the Methods section, and we therefore corrected the text to clarify that *w_{ij}* represents spatial weights (adjacency-based in our case), rather than distances. We also removed the sentence saying that the minimum and maximum values of Moran's I statistics are -1 and 1, which in general is not true. The statement can be approximate for perfectly regular lattices, but there is not a mathematical rule ensuring these are the lower and upper bounds. We clarified in the Methods that Moran's I typically takes positive values for clustered patterns, negative values for dispersed patterns, and values near zero for spatial randomness. See the updated lines 563-566.

Minor points:

- pg 2, lines 38, 41, 44: a bit of repetition of the "phenomenon/hallmark of lateral inhibition".

We have rewritten this part to avoid repetition (Lines 43-45).

- I could not find reference to figure 2c in the text

We added a reference to Fig. 2C in the main text (Line 87)

- Something appears to have gone wrong with the color bar of figure S4D.

We apologize for this. In the original submitted version the colors were correct, but something must have happened during the processing and formatting of the paper. In the new version the colormap should be fine.

- Are the magenta dot plots in S5 and 6 made with a single set of parameter values? If so, could you specify that in the legend - it was a bit hidden in the methods.

No, each magenta dot corresponds to a single pair of (gamma, alpha0) values taken from the parameter sweep detailed in the section "Parameter exploration" in the Materials and methods. Specifically, each magenta dot shown in the current Figs. S6 and S7 (former Figs. S5 and S6) corresponds to a pair of (gamma, alpha0) values depicted with gray dots in the associated phase diagrams (appearing in the panels above in Fig. S6 and on the left in Fig. S7). Magenta dots in Fig. S6B, D are the same as those shown in Fig. 3D, E.

We added a sentence in the captions of Figs. S6 and S7 to clarify this matter.

© 2026. Published by The Company of Biologists under the terms of the Creative Commons Attribution License (https://creativecommons.org/licenses/by/4.0/).

- ln 164: wonky sentence, probably just remove "what".

This mistake has been corrected, and another minor change has improved the sentence (Lines 181-182).

- Could you discuss whether you think that Jungermanniopsida retain the lateral inhibition mechanism, given the lack of species with scattered cells?

This is an interesting point. We currently do not know the specific genetic drivers of phenotypic differences in different liverwort branches. In the case of most Jungermanniopsida species, the presence of OBs in all cells could be a direct consequence of the loss of inhibitors (for instance, the loss of the TGA gene, a known inhibitor of OB differentiation in *M. polymorpha*). In that case, if TGA is the actual gene mediating lateral inhibition, one could say that the capacity for lateral inhibition has been lost in this branch. However, one would still need to explain why some branches in Jungermanniopsida (such as *Nowellia* and *Anthelia*) do not possess OBs, a fact that cannot be easily explained by the simple loss of an inhibitor—on the contrary, one would need to invoke an additional loss of activators.

Alternatively, the lack of scattered OBs could also be a consequence not of the direct loss of the inhibitor, but simply due to other factors (which can be either genetic, but also environmental) modulating the core activator-inhibitor network. If that were the case, lateral inhibition would still be present (in the sense that the inhibitor v can diffuse and inhibit the activator u in adjacent cells), but would not be not strong enough to generate a pattern-forming instability. The result would be an homogeneous state where all cells have OBs.

While given the current genetic knowledge we cannot distinguish between these two possibilities, the variation in OB cell distributions within the same liverwort branches suggest a flexibility that is easily achievable by the latter hypothesis, but would require multiple modifications of the regulatory network under the former. In other words, if the core lateral inhibition mechanism has been retained, transitions between scattered, ubiquitous, or absent OBs could emerge through small changes in the strength of activators, inhibitors, or through changes in environmental conditions. By contrast, if lateral inhibition had been lost through the loss of key inhibitory genes, the repeated emergence of different OB distributions would require additional evolutionary events affecting other parts of the network. Interestingly, Tanaka *et. al*. 2016 (cited in the manuscript) showed that this flexibility is already present within *M. polymorpha*, as plants grown under different conditions (agar vs. field grown) show dramatically different numbers of OB cells. Therefore, environmental modulation of a conserved patterning mechanism is sufficient to generate substantial variation in OB cell distributions, without requiring changes to the underlying gene network itself. Although this does not demonstrate that lateral inhibition has been retained in Jungermanniopsida, it suggests that the absence of scattered OB cells cannot be taken as evidence for the loss of the mechanism. Distinguishing between these alternatives would require comparative genetic analyses across different liverwort lineages, but such data is not yet available and therefore falls beyond the scope of this work.

To better clarify this point we added a section to the Discussion (Lines 215-225).

- In a similar vein, you mention in the discussion (line 198-205) that "the internal and spatial variability in OB cells is taxonomically important and could offer insights into the adaptive strategies of early land plants." Given that your model suggests that changes in patterning can result from straightforward shifts in parameters, how reliable would such a taxonomy be over longer evolutionary time scales?

We agree that our model implies that OB cell patterns can be sensitive to relatively small shifts in parameters and may therefore fluctuate over long evolutionary timescales. Our intention is not to suggest that specific pattern states constitute reliable phylogenetic markers, but that variation in OB cell organization remains taxonomically informative when interpreted in combination with other characters. We have rewritten this part to properly reflect this message (Lines 229-234).

© 2026. Published by The Company of Biologists under the terms of the Creative Commons Attribution License (https://creativecommons.org/licenses/by/4.0/).

- fig S9: Can you explain in the legend the choices for mutant parameters?

The caption of Fig. S10 (the former Fig. S9) has been updated to explain the parameter choices for the mutants. Thanks to this comment, we realized the black markers in panel B of Fig. S9 were not drawn in the locations corresponding to the parameters described in panel A. Therefore, we updated panel B in Fig. S9, correcting the locations of the black markers.

Second decision letter

MS ID#: dev.205696R1

MS TITLE: Lateral inhibition governs ancestral cellular patterning in fossil and extant liverworts

AUTHORS: Josep Mercadal, Susan Tremblay, Leonie Kraska, Martin A. Hutten and Pau Formosa-Jordan

Dear Dr Mercadal,

I am happy to tell you that your manuscript has been accepted for publication in Development, pending our standard publication integrity checks.

© 2026. Published by The Company of Biologists under the terms of the Creative Commons Attribution License (https://creativecommons.org/licenses/by/4.0/).
